# Safety Profiles of *Tripterygium wilfordii* Hook F: A Systematic Review and Meta-Analysis

**DOI:** 10.3389/fphar.2016.00402

**Published:** 2016-11-08

**Authors:** Chi Zhang, Ping-Ping Sun, Hong-Tao Guo, Yan Liu, Jian Li, Xiao-Juan He, Ai-Ping Lu

**Affiliations:** ^1^Institute of Basic Research in Clinical Medicine, China Academy of Chinese Medical SciencesBeijing, China; ^2^School of Chinese Medicine, Hong Kong Baptist UniversityKowloon, Hong Kong; ^3^School of Basic Medical Sciences, Beijing University of Chinese MedicineBeijing, China; ^4^The First Affiliated Hospital of Henan University of Traditional Chinese MedicineZhengzhou, China

**Keywords:** *Tripterygium wilfordii* Hook F, safety, adverse event, systematic review, meta-analysis

## Abstract

**Objective:** *Tripterygium wilfordii* Hook F (TwHF) is a widely used and effective treatment for inflammatory diseases. There have been concerns about its toxicity but no adequate synthesis of the evidence for adverse events (AEs). We aimed to undertake a clinically informative, systematic safety profile of TwHF.

**Methods:** We undertook a systematic review and meta-analysis of experimental studies and observational studies. We searched electronic databases and conference abstracts. Safety outcomes were rates of common AEs.

**Results:** We screened 4137 abstracts for eligibility and included 594 studies in the analysis. The overall incidence of AEs was 26.7% (95% CI 24.8%, 28.8%) in 23,256 TwHF users. The estimates did vary markedly when stratified by specific study types. The incidence of gastrointestinal symptoms, adverse reproductive outcomes, adverse skin reactions, hematologic events and cardiovascular events were 13.3% (95% CI 11.9%, 14.9%), 11.7% (95% CI 10.3%, 13.3%), 7.8% (95% CI 6.3–9.5%), 6.5% (95% CI 5.7–7.4 %) and 4.9% (95% CI 1.6 %, 14.3 %), respectively. The prevalence of irregular menstruation (IM) was increased in patients taking TwHF compared with those given control (odds ratio [OR] 4.65, 95% CI 3.08 to 7.03). TwHF use has lower risk of weight gain (OR 0.12 [95% CI 0.04 to 0.39]) and hair loss (OR 0.37 [95% CI 0.18 to 0.78]). Furthermore, long-term aspirin use (>6 months) has a higher AEs incidence (31.0% [95% CI 24.5%–38.5%]).

**Conclusion:** Our findings suggest that more than one in four patients who were taking TwHF had experienced AEs. A clear need exists for improved understanding of contributing risk factors, as well as of prevention and management strategies to improve patients' tolerance for TwHF.

## Introduction

*Tripterygium wilfordii* Hook F (TwHF), a member of the Celastraceae family, is a woody vine native to the south side of the Tangtze River, China. For more than a decade, experts from across the globe have been working on exploring the effect of TwHF. There have been many studies published on the potential use of this herb. As of June 1, 2016, about 200 journal articles related to TwHF had ten or more citations. These *in vitro* and *in vivo* studies confirm the therapeutic benefits of it. TwHF is used to be a novel immunosuppressive and anti-inflammatory agent, especially for rheumatoid arthritis (RA)(Tao et al., 2002; Goldbach-Mansky et al., 2009; Lv et al., 2015), nephrotic syndrome (Xu et al., 2009), diabetic nephropathy(Ge et al., 2013), and kidney transplantation(Ji et al., 2006). It has also been reported as an antitumour agent (Wong et al., 2012). In addition, reports discussed the role of TwHF in the treatment of HIV/AIDS (Duan et al., 2000) and male fertility regulation(Qian, 1987).

Although efficacious, TwHF has some safety disadvantages (Canter et al., 2006). Previous studies have demonstrated the adverse menstrual effects, and TwHF might be most appropriately used by patients who are postmenopausal or not interested in fertility (Qian, 1987). Furthermore, TwHF can impair liver and renal function and is not recommended for use in patients with decreased liver or renal function. In April 2012, the State food and drug administration (China SFDA) has issued the No 46 Adverse Drug Reaction Information Bulletin to *Tripterygium wilfordii* preparations users (http://www.sda.gov.cn/WS01/CL0078/70473.html; Accessed May 16, 2016). However, this bulletin did not provide sufficient information to clinicians. What is the incidence of adverse events (AEs) in TwHF users? What are the characteristics and risk factors of AEs? Published systematic reviews (SRs) usually put a strong emphasis on efficacy and fail to investigate safety (Chen et al., 2010; Ernst, 2011). Randomized controlled trials (RCTs) have offered solace for practitioners and their patients, relatively common adverse effects vs. methotrexate (MTX) (Lv et al., 2015). Inconsistency between observational studies and RCTs confuses researchers and should be carefully interpreted (Canter et al., 2006). Whatever, the history of long-term use does not well establish the safety of TwHF.

Due to its anti-inflammatory and immunosuppressive effects it may be comparable with MTX (Lv et al., 2015), TwHF may be considered as an alternative or complementary therapy for treatment of RA or other inflammatory diseases. It might lead to wider use of TwHF. Clinicians and their patients therefore need accurate evidence of harms. In this study, we performed a comprehensive analysis of the current available evidence to estimate the incidence of AEs and investigate the association between TwHF use and reported major AEs, to provide a clinically informative safety profile for TwHF.

## Methods

### Study protocol and search strategy

The study protocol has been registered with the International Prospective Register for Systematic Reviews (PROSPERO) CRD 42014010186. This work followed the methods specified in the Cochrane Handbook for Interventional Systematic Reviews (Higgins et al., 2008). The Preferred Reporting Items for Systematic Reviews and Meta-Analyses (PRISMA) statement was used as a basis for reporting this work (Supplementary File 1). We searched Pubmed, Embase, China National Knowledge Infrastructure (CNKI), Wanfang, VIP, and Sionmed from their inception to 16 January 2016 (Supplementary File 2). No language restriction was applied. All relevant references were checked for additional citations. Major conference abstracts were hand-searched.

### Study selection

Abstracts and articles titles were reviewed by pairs of authors (CZ and PPS), working independently. Once the articles were chosen, inclusion was discussed by all authors to resolve differences of opinion. Studies contained original data were included if they investigated one or more AEs of interest. RCTs and controlled clinical trials (CCTs) comparing TwHF with placebo, no treatment, or other drug therapies were considered most reliable if they included safety data, followed by cohort studies and then case-control studies. In the absence of controlled studies, we included prospective single arm studies (PSSs) following up patients given TwHF and, finally, case series and individual case reports. For redundant publication, we analyzed only the most complete set of data to avoid double counting cases.

### Outcomes

The safety outcomes of this study were incidences of AEs. The known AEs includes gastrointestinal symptoms, adverse reproductive outcomes, cardiac damage, kidney and liver damage, skin disorders, hair disorders, weight change and even death. Incidence of AEs = (number of patients with AEs/total patients studied) × 100%.

### Data collection

Data was abstracted independently by two authors (CZ and PPS) using data extraction forms (Supplementary Files 3–7). All disagreements were resolved through discussion with a third reviewer (APL). The following variables were recorded: study name, first author, date of publication, disease, interventions, number of patients, duration of follow-up, age, gender, quality aspects and all AEs profiles. For safety endpoints, we extracted the number of events of interest and total number of patients in each group.

### Quality assessment

Quality assessment was also independently performed by two researchers (CZ and PPS), and disagreements were resolved by consensus. Cochrane Collaboration's risk of bias tool was used to access the methodological quality of the included RCTs.

### Statistical analysis

When appropriate, data from individual prospective studies were pooled by meta-analysis with Metaanalyst Beta 3.13 and STATA 11.1 (Stata Corp, College Station, TX). We analyzed the incidence of AEs obtained from different studies to determine the meta-analytic weighted average and the 95% confidence interval (CI). We used a random-effects model to perform the analysis in order to take into account the heterogeneity of the studies. The overall incidence was estimated as a weighted average, iteratively calculating the weights and the heterogeneity variance they depend upon. The odds ratios (ORs) compare the likelihood of an AE between two groups. Heterogeneity between study-specific estimates was investigated. Sensitivity analysis was carried out to explore the effect of trial quality on overall results. Descriptive method was applied to summarize the adverse outcomes of TwHF for case series and case reports.

## Results

### Study characteristics

**Figure 1:**
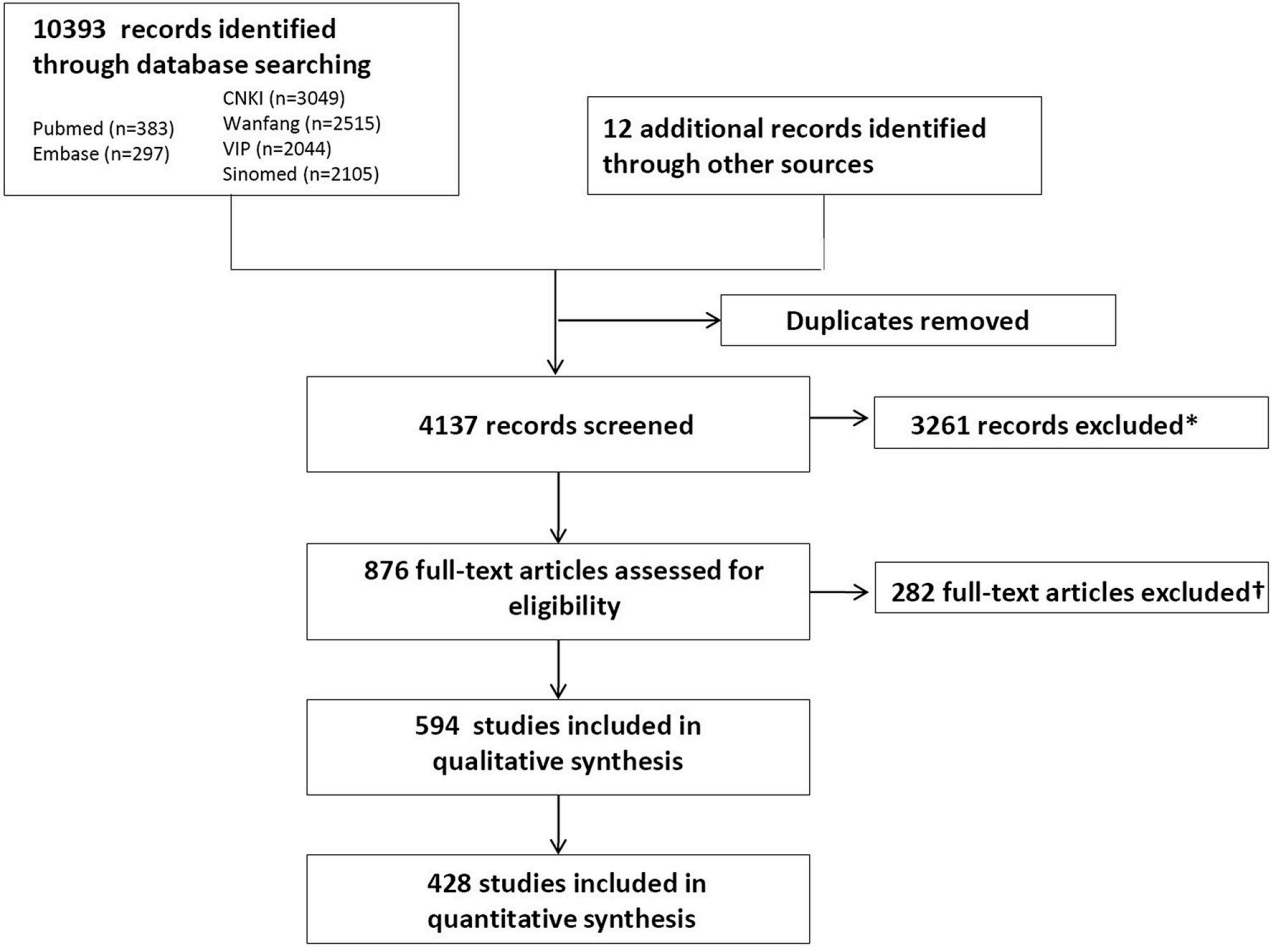
**PRISMA (Preferred Reporting Items for Systematic Reviews and Meta-Analyses) flowchart of the systematic literature search**. ^*^Included animal studies, non-biological science studies, and human studies of *Tripterygium wilfordii* Hook F (TwHF) not reporting adverse events (AEs). ^†^Included 108 with no records for AEs; 71 articles with TwHF as basic treatment; 31 research allocated the patients into different groups through registration number and 2 through pathological type but all of them were rated as random trials; 37 duplicate publication of the same research; 7 articles with problems in the reporting like inconsistent data; 12 plagiarized articles; 9 irrelevant and 5 articles with unclear source of the samples.

After screening 4137 citations, we reviewed 876 potentially relevant full-text articles and identified 594 primary publications (264 RCTs, 100 CCTs, 156 PSSs, 8 case series and 66 case reports) (Figure 1) (Supplementary File 8). Baseline characteristics of individual studies are summarized in Supplementary File 3–7. In total, 48,536 patients were included in the qualitative analysis.

264 RCTs recruited altogether 27,368 patients, with the smallest sample size 13 and the largest size 3789. In 206 studies, TwHF was given to experimental group or both experimental and control group, which included 17,265 patients. In the remaining 58 clinical studies, TwHF was allocated to control. In addition, 100 CCTs recruited 10,503 patients, the sample size ranges from 12 to 656. In 81 of 100 studies, TwHF was given in active group. Totally 10,441 patients were included in 156 uncontrolled PSSs, and sample size from 4 to 606. Eight case series reported 127 patients, and 66 case reports reported 97 cases.

The quality of evidence varied according to the Cochrane Collaboration's tool. The majority of the included studies had unclear risk of bias (Supplementary File 9. Risk of bias graph). Studies published in English, and Chinese were included. No study in third language identified met inclusion criteria. The research sittings were covering 94% of provincial level regions (29/31, 22 provinces, four autonomous regions, and four municipalities, except Tibet and Qinghai) in mainland China. One multicenter study was done in 11 centers of the United States (Goldbach-Mansky et al., 2009).

**Figure 2:**
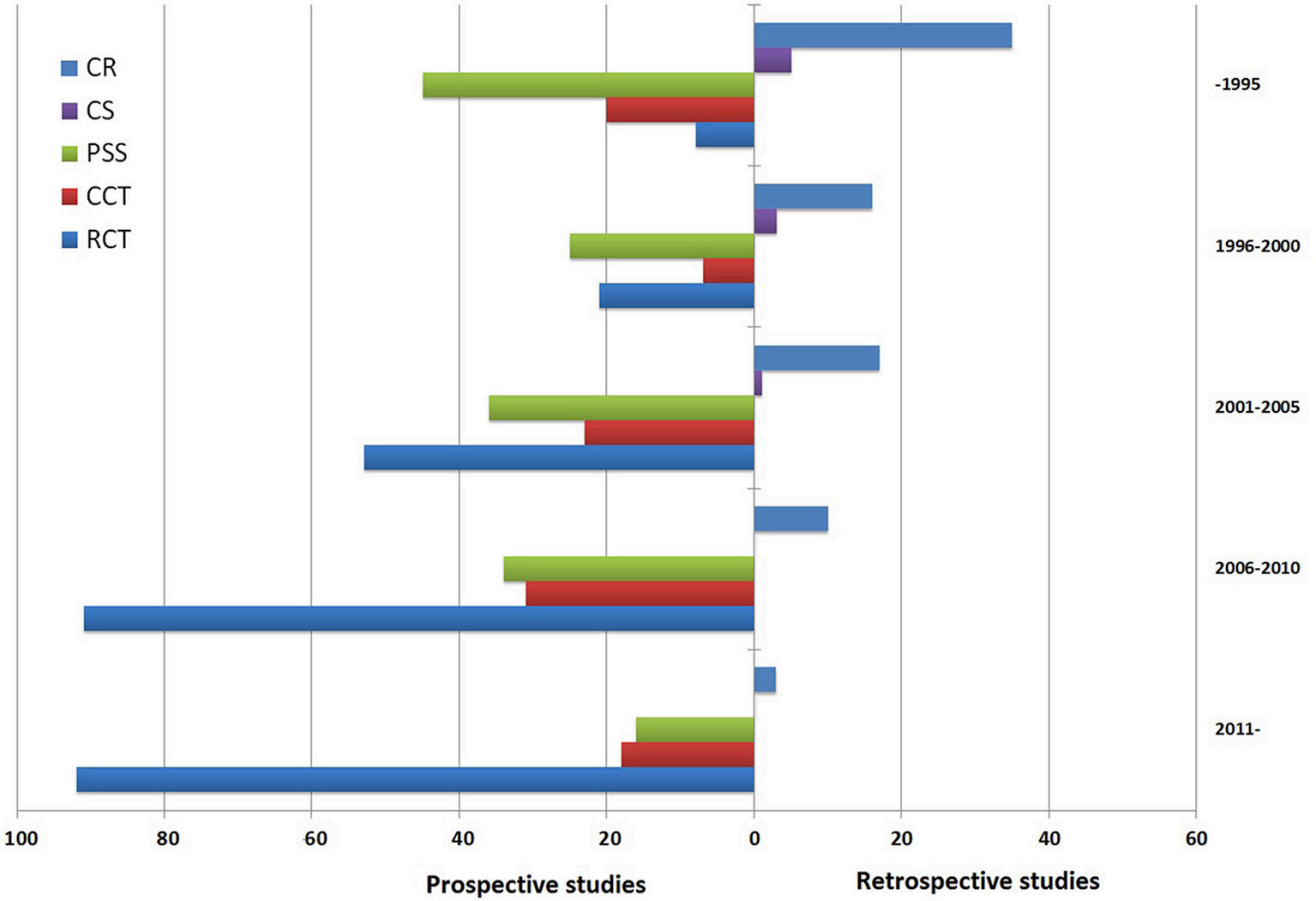
**Time trend of 594 articles relevant to ***Tripterygium wilfordii*** Hook F (TwHF) adverse events (prospective studies vs. retrospective studies)**. The search was performed on March 2016. RCT, randomized controlled trial; CCT, controlled clinical trial; PSS, prospective single arm study; CS, case series; CR, case report.

Considering a possible source of bias might the year of study, we did an initial analysis of publication time trends. Figure 2 shows the results graphically and indicates that clinical study design change occurred during 36-year span. This result seems surprising since great changes have occurred over the last decades in case series and case reports that nearly disappear. Perhaps, RCTs are more likely to be accepted by the journals. Therefore, when analyzing the association between TwHF and all reported major AEs, we need to consider the influence of specific study types for risk estimate.

### Safety outcomes

Generally, 428 studies presented 23,256 patients with 7616 events. 273 studies reported 2469 gastrointestinal upsets, 228 articles pointed 1663 adverse reproductive outcome, 79 papers presented an increase of ALT was observed among 476 patients, 43 articles reported 193 events related to renal injury, 250 articles reported 773 adverse hematologic events, 10 papers with 33 cardiovascular events. 108 studies accounted for 640 skin and mucosa AEs and 159 articles reported other events include hair loss, weight loss and fatigue which amount to 1007.

**Figure 3:**
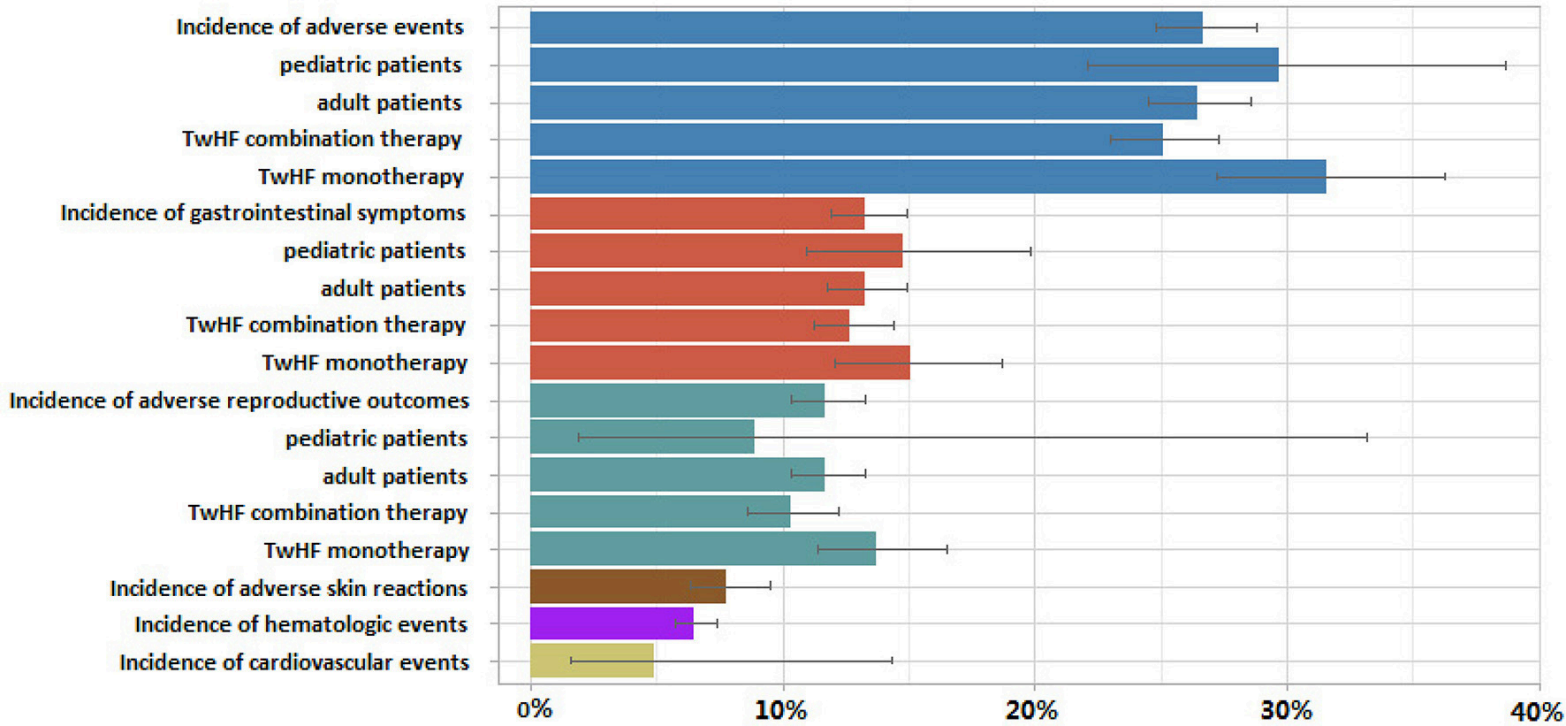
**Random-effect pooled estimates of incidence of total adverse events, gastrointestinal symptoms, adverse reproductive outcomes and their subsequent subgroup analyses, along with adverse skin reactions, hematologic events and cardiovascular events after ***Tripterygium wilfordii*** Hook F (TwHF) therapy**. Each color represents a different type of adverse event.

As shown in Figure 3, the weighted proportion of AEs was 26.7% (95% CI 24.8%, 28.8%) among patients with TwHF. The results were the same in leave-one-out sensitivity analysis. 29.7% (95% CI 22.1%, 38.7%) of pediatric patients and 26.5% (95% CI 24.5%, 28.6%) of adults experienced AEs. The incidence of AEs among combination therapy studies (25.1% [95% CI 23.0%, 27.3%]) was lower than among monotherapy studies (31.6% [95% CI 27.2%, 36.3%]). The rate of AEs were 23.6% (95% CI 21.2%, 26.2%), 29.9% (95% CI 24.9%, 35.4%), 31.6% (95% CI 27.6%, 35.9%) among RCT, CCT, PSS designs studies. Results suggest that the hierarchy of evidence could influence the incidence rate of TwHF AEs. The role of TwHF in the trials, as a treatment in interventional or control group, may be a potential risk factor. Our estimate of AEs incidence was 21.8% (95% CI 19.1% to 24.9%), 29.8% (95% CI 25.4% to 34.6%), 29.1% (95% CI 23.5% to 35.3%) and 32.7% (95% CI 22.7% to 44.4%) when restricted to data from RCT treatment group (30 studies), RCT control group (30 studies), CCT treatment group (30 studies), CCT control group (30 studies), respectively.

In another subgroup analysis, the reported gastrointestinal symptoms incidence was 13.3% (95% CI 11.9%, 14.9%), 14.8% (95% CI 10.9%, 19.8%), 13.3% (95% CI 11.8%, 14.9%) of total population, pediatric patients and adult patients respectively. Meta-analysis showed gastrointestinal symptoms occur less frequently with TwHF combination therapy (12.7% [95% CI 11.2% to 14.4%]) than in patients with monotherapy (13.8% [95% CI 12.1% to 15.7%]). In RA patients, the combined rate of all gastrointestinal symptoms was significantly lower in TwHF combination therapy-treated patients than in conventional treatment-treated patients (OR = 0.59, 95% CI, 0.46 to 0.76; *P* < 0.001).

Total reproductive adverse events (RAE) incidence was 11.7% (95% CI 10.3%, 13.3%) among TwHF users. Concerning specific issues dealing with the safety of TwHF in children, six studies examined RAE occurrence in minors. RAE incidence was 8.9% (95% CI 1.9% to 33.2%) and 11.7% (95% CI 10.3% to 13.3%) of pediatric and adult patients. Forty-four studies (*n* = 1207) reported 133 cases of irregular menstruation (IM), IM incidence was 12.7% (95% CI 10.1 to 15.8%). The prevalence of IM was increased in patients taking TwHF compared with those given control (OR 4.65, 95% CI 3.08 to 7.03; *P* < 0.001).

Ten studies reported cardiovascular events occurrence in 555 TwHF users. The rate of cardiovascular events was 4.9% (95% CI 1.6%, 14.3%). The meta-analytical yielded the frequency of ALT elevation cases (greater than abnormal liver function) of 4843 patients in 78 studies was 8.6% (95% CI 6.8–10.7%). Incidence of hematologic events and adverse skin reactions was 6.5% (95% CI 5.7–7.4%) and 7.8% (95% CI 6.3–9.5%), respectively.

Five studies reported weight changes, include 3 RCTs, 1 CCT and 1 PSS. Compared to Prednisone (3 studies, *n* = 129), TwHF use was associated with reduced weight gain incidence risk (OR 0.12 (95% CI 0.04 to 0.39; *P* = 0.001; heterogeneity *I*^2^ = 0). One PSS (*n* = 13) suggested 1 case of weight loss only in TwHF group whereas one CCT (*n* = 52) suggested 2 case of weight loss in MTX group and no weight loss case in TwHF monotherapy or combination therapy group.

Thirty-eight publications reported an AE of TwHF on hair loss, 8 of which were case reports, 7 of which were PSSs. 15 RCTs of TwHF (n = 383) vs. control (n = 402) reported hair loss in eleven of 383 (2.9%) patients in the TwHF group compared with 38 of 404 (9.4%) in the control group. 8 CCTs reported hair loss in one of 263 patients given TwHF vs. 13 of 309 given control treatment. The incidence of hair loss was lower with TwHF than with control treatment (*n* = 1357; OR 0.37, 95% CI 0.18 to 0.78, *P* = 0.012; heterogeneity *I*^2^ = 25%).

**Figure 4:**
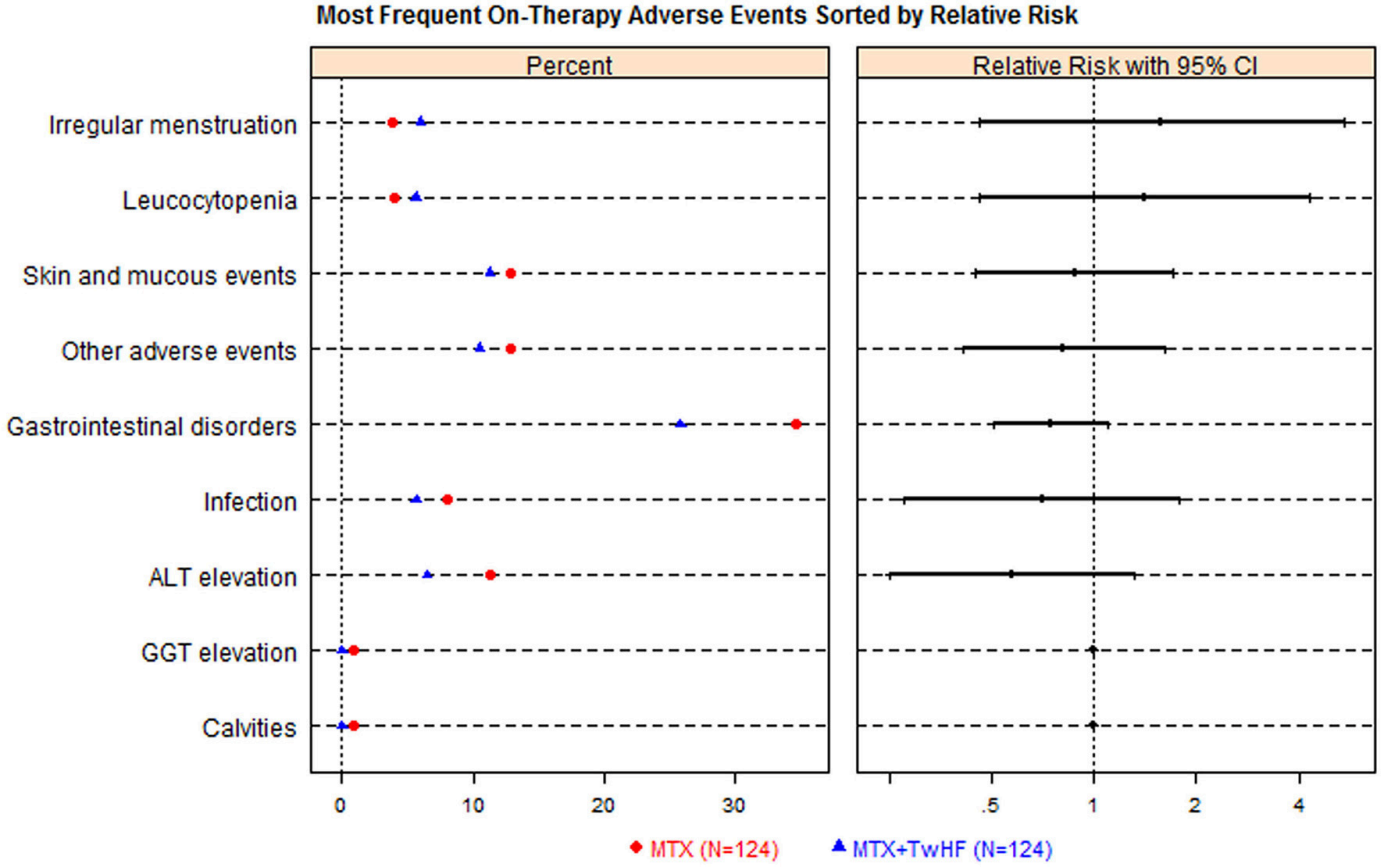
*****Tripterygium wilfordii*** Hook F (TwHF) plus methotrexate (MTX) vs. methotrexate (MTX) alone adverse events dot plot with relative risk (***n*** = 248)**. The right hand panel displays a relative risk plot, graphically presenting the relative risks, with associated 95% conference intervals, these helping to determine risk relative to other signals.

TwHF users with treatment period of 6–12 months were always exhibit the relatively high occurrence of AEs than the shorter-term users (Table 1), all types of AEs other than adverse skin reactions. Two RCTs (*n* = 248) compared AEs between TwHF plus MTX with MTX monotherapy (Figure 4). The combination group was greater than MTX group in the incidence of gastrointestinal disorders, skin reaction, ALT elevation and infection, but failed in leucocytopenia and irregular menstruation.

**Table 1:** **Incidence of adverse events of ***Tripterygium wilfordii*** Hook F and the length of treatment period**.

	**Incidence of adverse events (%; 95% CI)**	**Incidence of gastrointestinal symptoms (%; 95% CI)**	**Incidence of adverse reproductive outcomes (%; 95% CI)**	**Incidence of adverse skin reactions (%; 95% CI)**	**Incidence of ALT elevation (%; 95% CI)**
≤3 (months)	23.4 (20.6–26.3)	13.2 (11.1–15.6)	9.6 (8.0–11.6)	8.8 (6.4–11.9)	7.3 (5.3–10.0)
3 < Time ≤ 6 (months)	26.3 (21.6–31.6)	13.2 (10.2–16.9)	12.5 (9.1–16.9)	8.2 (5.3–12.5)	9.1 (5.9–13.8)
6 < Time ≤ 12 (months)	31.0 (24.5–38.5)	19.2 (14.4–25.3)	13.5 (8.5–20.8)	5.4 (3.1–9.0)	11.1 (2.9–34.0)
12 < Time (months)	31.2 (22.0–42.3)	13.3 (9.2–18.9)	7.8 (4.3–13.7)	4.4 (1.0–17.4)	4.8 (0.7–27.1)

### Case series and case reports

All case reports and case series were from mainland China. Sixty-six case reports included 49 females and 46 males, and 2 unclear cases (Supplementary File 7). There were 7 cases less than or equal to the age of 18 years old, 15 cases older than 60 years old. Of 97, death occurred in 9 patients following an overdose of TwHF. Ten reports presented 10 cases with organ failure, including 6 cases with acute renal failure and 4 cases of TwHF-induced premature ovarian failure. Seven papers reported 11 cases of aplastic anemia. Four articles reported skin rashes, including 2 cases of definite drug-induced rashes, 1 with allergic urticarial and 1 with erythra. Three articles reported hair loss, including 2 cases with diffuse hair loss and 1 temporary hair loss. In case series, TwHF overdose in 59 patients with renal failure were described. However, it is worth mentioning that 17 people who attempted suicidal out of 59 patients are from remote rural areas of western China which are likely to die with fresh root portion of TwHF plant. It is also noteworthy that, the incidence of fatal accident in past 10 years was the lowest in recorded history.

## Discussion

To date, this is the most comprehensive analysis that specifically deals with clinical safety of TwHF. This study, focusing on the incidence, known or potential risk factors of TwHF AEs, is aimed at informed clinical decision-making. Our findings suggest that 26.7% TwHF users experienced AEs. These findings are supported by evidence from 428 prospective studies.

A stratified analysis provides additional insight for known or potential risk factors of TwHF AEs. First, the incidence of TwHF AEs seems to increase over time, after TwHF therapy (Table 1), although pooled estimates have shown that cases continue to accumulate beyond this period but at a slower rate. Second, there is substantial evidence to suggest that exposure to children and monotherapy enhance the risk of AEs. These factors are potential targets for the prevention of adverse outcomes. Finally, as shown in Figure 2, the incidences of AEs are significantly affected by study type. However, it is different from the results of a methodological study which suggests that study type might have no effect on AE incidence (Golder et al., 2011). Whether a greater number of events are hidden by an inappropriate reading of significance testing in RCTs? Clinicians and researchers must consider the study design when interpreting and designing RCTs of TwHF.

Results have suggested that TwHF may increase risk of reproductive toxicity, adverse gastrointestinal complications, and abnormal liver function (Figures 3, 4). According our findings (Table 1), TwHF should not be used for prolonged periods of time to increase risk of adverse reproductive outcomes. Despite the availability of newer biological agents, RA patients were at a higher risk of GAEs than the general population (Salliot and van der Heijde, 2009). Combined results showed less GAEs in TwHF combination therapy group than in MTX monotherapy groups. TwHF combination therapy may enhance tolerability because they reduce gastrointestinal toxicity of TwHF monotherapy. Hence, the potential applications of combination therapy can provide powerful clues as to the role in reducing harms. Our study indicated the frequency of ALT elevation (more than normal) in TwHF users was 8.6% (95% CI 6.8–10.7%). A meta-analysis suggested the prevalence of elevated liver enzymes (more than twice upper limit of normal) is close to 13% of the patients with MTX (Salliot and van der Heijde, 2009). Uncertainties exist due to the limited amount of data in some of the other enzymes found the liver (such as AST). In our included literatures, no death was reported in prospective studies. All nine cases of overdose mortality were from case reports. Totally, the potential determinants of overdose death are both poorly understood and studied.

Data on the risk for body weight have further questions. A study, published in the journal Cell (Liu et al., 2015), found that an extract in TwHF has a huge weight loss effect on obese mice. However, animal experiment and clinical trial seem to offer contradictory evidence on whether TwHF contribute to mice weight loss or weight gain (Wang et al., 2000; Tao et al., 2001). There are several factors might explain the association between TwHF and weight loss. GAEs include nausea, vomiting, loss of appetite, or diarrhea. Pooled data of human subject's studies provides support for the hypothesis that TwHF use could reduce weight gain risk compare with Prednisone which is often used in RA patient treatment. These results should be carefully interpreted and need to be explored through further research.

## Limitations

The main limitation of this study is highly variable in study types. 594 studies were published over the entire period from 1979 to 2015 (Figure 2). Diagnostic criteria, treatments and accuracy of measurements of physiological parameters have changed during that period. Although we find combination therapy has potential advantages, the determination of the heterogeneity between drugs combination is still a question in meta-analyses (Safdar et al., 2004). We deal with heterogeneity among the studies in numerous ways: (1) we used a random-effects model to do the analysis because it takes into account the heterogeneity of the various studies, (2) we excluded studies with unclear information, (3) and we also excluded retrospective data in combined analysis. But even so, we cannot be certain that the heterogeneity in the intervention groups and the control groups did not influence the estimates. In our study, only under the appropriate conditions, we combined to produce ORs.

Moreover, because most CCTs and RCTs did not use a patient group that was new to TwHF (or did not provide this information), length of follow-up was usually poorly defined so the average interval between first starting TwHF and the onset of AEs is unknown. Furthermore, we could not obtain any exact individual information of TwHF from China SFDA. According to the national adverse drug reaction monitoring center case report data, from Jan 2004 to Sep 2011, involving TwHF case report 839 cases, serious in 73. Our analysis suggested different incidences of AEs between controlled trial arms. The incidences were always lower in treatment groups than in control arms. Another limitation is the absence of the long-term cohort studies. Dose information was incompletely reported and any potential predictors of AE risk could not be specifically addressed in meta-analysis.

Also, most studies did not provide appropriate information of the number of TwHF specific AEs. For example, authors usually reported gastrointestinal AE but did not provide information about nausea or vomiting. Another unavoidable problem is that most of included primary studies come from Chinese journals. The major Chinese herbal researches are in China, and the many original research results are mainly published in Chinese. Since global researchers demand for TwHF relevant resources, regardless the languages, then the data from China needs to be included in the analysis. Compared with other Chinese herbal medicines, TwHF has been explored in many excellent international studies. Nonetheless, we were able to locate a reasonable amount of evidence that allows cautious conclusions to be drawn about the safety of TwHF.

## Conclusion

Our findings suggest that slightly more than one-fourth of patients who were taking TwHF had experienced AEs. A clear need exists for improved understanding of contributing risk factors, as well as of prevention and management strategies to improve patients' tolerance for TwHF. The effect estimates, varying by specific study types, deserve further investigation.

## Author contributions

Designers: CZ and AL; Searchers: CZ and PS; Studies appraisers: CZ, PS and all authors; Data extractors: CZ, PS, and HG; Analyzers: CZ, YL, and JL; Writers: CZ, XH, and AL.

### Conflict of interest statement

The authors declare that the research was conducted in the absence of any commercial or financial relationships that could be construed as a potential conflict of interest.

## Abbreviations

AE: adverse event
CCT: controlled clinical trial
MTX: methotrexate
OR: Odds ratio
PSS: prospective single arm study
RA: rheumatoid arthritis
RCT: randomized controlled trial
SR: systematic review
TwHF: *Tripterygium wilfordii* Hook F.
