# Corrigendum: Safety Profiles of *Tripterygium wilfordii* Hook F: A Systematic Review and Meta-Analysis

**DOI:** 10.3389/fphar.2017.00059

**Published:** 2017-02-15

**Authors:** Chi Zhang, Ping-Ping Sun, Hong-Tao Guo, Yan Liu, Jian Li, Xiao-Juan He, Ai-Ping Lu

**Affiliations:** ^1^Institute of Basic Research in Clinical Medicine, China Academy of Chinese Medical SciencesBeijing, China; ^2^School of Chinese Medicine, Hong Kong Baptist UniversityKowloon, Hong Kong; ^3^School of Basic Medical Sciences, Beijing University of Chinese MedicineBeijing, China; ^4^The First Affiliated Hospital of Henan University of Traditional Chinese MedicineZhengzhou, China

**Keywords:** *Tripterygium wilfordii* Hook F, safety, adverse event, systematic review, meta-analysis

In the original article, there was an error. **“Aspirin” was referred to instead of “TwHF.”**

A correction has been made to **the last sentence of the Results section in the abstract**:

We screened 4137 abstracts for eligibility and included 594 studies in the analysis. The overall incidence of AEs was 26.7% (95% CI 24.8%, 28.8%) in 23,256 TwHF users. The estimates did vary markedly when stratified by specific study types. The incidence of gastrointestinal symptoms, adverse reproductive outcomes, adverse skin reactions, hematologic events and cardiovascular events were 13.3% (95% CI 11.9%, 14.9%), 11.7% (95% CI 10.3%, 13.3%), 7.8% (95% CI 6.3–9.5%), 6.5% (95% CI 5.7–7.4 %) and 4.9% (95% CI 1.6 %, 14.3 %), respectively. The prevalence of irregular menstruation (IM) was increased in patients taking TwHF compared with those given control (odds ratio [OR] 4.65, 95% CI 3.08 to 7.03). TwHF use has lower risk of weight gain (OR 0.12 [95% CI 0.04 to 0.39]) and hair loss (OR 0.37 [95% CI 0.18 to 0.78]). Furthermore, long-term TwHF use (>6 months) has a higher AEs incidence (31.0% [95% CI 24.5%–38.5%]).

The authors apologize for this error and state that this does not change the scientific conclusions of the article in any way.

## Conflict of interest statement

The authors declare that the research was conducted in the absence of any commercial or financial relationships that could be construed as a potential conflict of interest.

