# Supplementary material for: Safety Profiles of Tripterygium wilfordii Hook F: A Systematic Review and Meta-Analysis
Source: Front Pharmacol. 2016 Nov 8;7:402. doi: 10.3389/fphar.2016.00402 (PMC5099241; doi:10.3389/fphar.2016.00402)
Supplement: Supplementary file 2 [file DataSheet2.doc]

**Additional file 1** **Example search strategy to identify studies from electronic databases.**

**PUBMED** (http://www.ncbi.nlm.nih.gov/pubmed/)

(((((((((((((((((((tripterygium) OR tripterygium) OR tripterygium wilfordii) OR tripterygium wilfordii hook.f) OR tripterygium wilfordii) OR wilfordii, tripterygium) OR wilfordii, tripterygium) OR thunder god vine) OR thunder god vine) OR vine, thunder o) OR vines, thunder o) OR leigong teng) OR leigong tengs) OR teng, leigong) OR tengs, leigong)) OR lei gong teng) OR lei gong teng)) AND (((((((((((((((((((adverse event) OR adverse events) OR adverse effect) OR adverse effects) OR adverse reaction) OR adverse reactions) OR safety) OR side effects) OR side effect) OR toxic) OR toxicity) OR toxic reaction) OR impair) OR damage) OR injury) OR harm) OR undesirable effects) OR injurious effects) OR poisoning)

**CNKI** (http://www.cnki.net/)

( ( ( 主题=雷公藤 OR 题名=雷公藤 ) OR 关键词=雷公藤 ) OR 摘要=雷公藤 ) AND ( 关键词=毒性 OR 关键词=不良反应 OR 关键词=副反应 OR 关键词=损害 OR 关键词=损伤 OR 关键词=危害 OR 关键词=毒害作用 OR 关键词=毒副作用 OR 关键词=毒副反应 OR 关键词=副作用 OR 摘要=毒性 OR 摘要=不良反应 OR 摘要=副反应 OR 摘要=损害 OR 摘要=损伤 OR 摘要=危害 OR 摘要=毒害作用 OR 摘要=毒副作用 OR 摘要=毒副反应 OR 摘要=副作用 )
