# Supplementary material for: Safety Profiles of Tripterygium wilfordii Hook F: A Systematic Review and Meta-Analysis
Source: Front Pharmacol. 2016 Nov 8;7:402. doi: 10.3389/fphar.2016.00402 (PMC5099241; doi:10.3389/fphar.2016.00402)
Supplement: Supplementary file 8 [file DataSheet8.doc]

**References**

**Randomized controlled trials**

1. Bao LX. Desloratadine plus Tripterygium glycosides for chronic urticarial. China Journal of Leprosy and Skin Diseases, 2008, 24(7): 568-569.
2. Bao Y. Clinical observation of Tripterygium glycosides combined with the small and middle dose of prednisone in the treatment of senile primary nephrotic syndrome. The Asia-pacific Traditional Medicine, 2013, 9(7): 172-173.
3. Cai H, Meng M, Xu SG, et al. Compare Zhengqing Fengtongning with Tripterygium glycosides for IgA nephropathy. Clinical Journal of Medical Officer, 2010, 36(5): 690-691.
4. Cai YB, Zheng GH, Zhou XW. Combined Tripterygium glycosides with prednisone for adult purpura nephritis. Chinese Community Doctors, 2012, 14(26): 99-100.
5. Cao S, Chen QP. Tripterygium glycosides tablets plus Valsartan for IgA nephropathy. Practical Clinical Medicine, 2013, 13(11): 9-10.
6. Chang GX, Wen CD. Tripterygium glycosides in the treatment of purpura nephritis. Proceeding of Clinical Medicine, 2009, 18(11): 841-842.
7. Chang J. Zhang WJ, Song XQ, et al. Combined leflunomide with Tripterygium glycosides for IgA nephropathy: a controlled trial. China Practical Medical, 2011, 6(25): 9-10.
8. Chen FY, Yu GY, Wang H. Tripterygium glycosides tablets plus Xin huang tablets in the treatment of rheumatoid arthritis patients with syndrome of stagnant dampness-heat. Clinical Journal of Chinese Medicine, 2010, 2(22): 86-87.
9. Chen GZ, Lin HQ, Chen GP. Qinzhi decoction for 52 cases of rheumatoid arthritis. Shanxi Journal of Traditional Chinese Medicine, 2005, 21(4): 13-14.
10. Chen HM. Tongbi Leigongteng decoction plus Maqian capsule for Rheumatoid arthritis: 39 cases clinical study. Journal of Practical Traditional Chinese Medicine, 2009 (8): 514-515.
11. Chen HM. Tongbi Tufuling decoction for acute gouty arthritis: 80 cases clinical study. Journal of Practical Traditional Chinese Medicine, 2009 (8): 514-515.
12. Chen KQ. Low-dose mifepristone combined with Tripterygium glycosides for hysteromyoma. Journal of Clinical Research, 2009, 26(7): 1185-1187.
13. Chen P, Zhu L, Zou R, et al. Tripterygium glycosides plus methotrexate for the treatment of rheumatoid arthritis: a randomized controlled trial. Journal of Anhui Traditional Chinese Medical College, 2011, 30(6): 28-32.
14. Chen WW. Clinical observation of Tripterygium glycosides combined with the middle dose of prednisone in the treatment of senile primary nephrotic syndrome. World Journal of Integrated Traditional and Western Medicine, 2013, 8(10): 1031-1033.
15. Zhan X, Li H. Clinical trial of Zhengqing Fengtongning for the treatment of rheumatoid arthritis. Journal of Anhui Traditional Chinese Medical College, 2005, 24(1): 9-11.
16. Chen XJ, Gu JH, Hu QR, et al. Clinical controlled study on treatment of active rheumatoid arthritis with Yiqi Qingluo Decoction. Acta University Traditional Medical Sinensis Pharmacologiaeque Shanghai, 2006, 19(4): 25-27.
17. Chen Y, Cao JJ, Yang WJ. 40 Cases with rheumatoid arthritis treated by electroacupuncture and warm moxibustion. Journal of Traditional Chinese Medicine, 2011, 26(6): 1173-1174.
18. ChenY, Tan Y. Clinical study of Triple therapy for IgA nephropathy. Modern Medicine & Health, 2009, 25(11): 1645-1646.
19. Chen Y. Treatment of idiopathic membranous nephropathy with prednisone combined with cyclophosphamide, tripterygium wilfordii or leflunomide：A prospective randomized controlled trial. China Medical University, 2013.
20. Chen ZM, Chen ZJ. Clinical study of 36 cases of refractory nephrotic syndrome treated with large dose Tripterygium glycosides. Chinese General Practice, 2003, 6(11): 948.
21. Chen Z, Tu SH, Hu YH, et al. A randomized controlled clinical trial of Chinese medicine vs western medicine in the treatment of rheumatoid arthritis. Chinese Journal of Rehabilitation, 2011, 26(6): 415-417.
22. Chen ZS, Weng CH, Lai YH. Efficacy of Tripterygium wilfordii combination therapy for 24 cases with nephrotic syndrome. The Chinese and foreign health abstract The Chinese and foreign health abstract, 2013 (33).
23. Chi XL. Clinical observation of Tripterygium glycosides for the treatment of diabetic nephropathy. Linchuang Heli Yongyao Zazhi, 2013, 6(23): 46-47.
24. Deng GZ. Efficacy and Safety of Different Dosage Multi-Glycosides of Tripterygium Wilfordii Hook. F. with Chinese Medicine for Hematuria in Children with Henoch-Schönlein Purpura Nephritis. Henan University of Traditional Chinese Medicine, 2007.
25. Deng ZZ, Lin YC, Ou RM, etal. Clinical observation of Tripterygium Wilfordii Hook. F. liposomes chitosan for the treatment of active rheumatoid arthritis. Journal of Guangzhou University of Traditional Chinese Medicine, 1998, 15(3):180-183.
26. Deng ZZ, Chen W. Tripterygium Wilfordii Hook. F. liposomes chitosan for the treatment of rheumatoid arthritis: a controlled trial. Journal of Traditional Chinese Medicine, 1998, 39(2):90-92.
27. Mo ZB. Efficacy of Tripterygium Glycosides plus antiviral pill for the treatment of 38 cases with pityriasis rosea. Contemporary Medicine, 2010 (13): 143-143.
28. Diao JN, Wang YY, Dong P. Tripterygium Glycosides and Fosinopril for CRP and TNF-αof patients with diabetic nephropathy. Chinese Journal of Integrated Traditional and Western Nephrology, 2013 (12): 1088-1089.
29. Dou BL. Combination treatment of Tripterygium glycosides and valsartan for patients with renal proteinuria. Public Medical Forum Magazine, 2010, 14(20): 672-672.
30. Dou YF. The clinical observation of Different Dosage Multi Glycosides of Tripterygium Wilfordii Hook. F. with Chinese Medicine for proteinuria in in Children with HSPN. Henan University of Traditional Chinese Medicine, 2007.
31. Du XF, Gao Y. Efficacy of Huangkui capsule combined with small dose of tripterygium glycosides in treatment of proteinuria in diabetic nephropathy. Chinese Journal of Integrated Traditional and Western Nephrology, 2011, 12(9): 822-823.
32. Du X. Clinical observation of Tripterygium glycosides combined with methylprednisolone for senile nephrotic syndrome. Jilin Medical Journal, 2012, 33(2): 283-284.
33. Du XL, Zhang ZL. Clinical trial of Lei luo zhi capsule for rheumatoid arthritis. Chinese Journal of Integrated Traditional and Western Medicine, 1998, 18(2): 88-91.
34. Duan DM, Cui MC. Clinical observation of urokinase combined with Tripterygium glycosides for the treatment of renal hematuria. China Medicine and Pharmacy, 2012, 2(19): 59-60.
35. Fan FR. Clinical efficacy of Ebastine Combined with Tripterygium Glycosides in the treatment of chronic urticarial. The Journal of Medical Theory and Practice, 2013, 26(8): 993-994.
36. Fei LS, Liu JJ. Tripterygium Glycosides and transfer factors for the treatment of rheumatoid arthritis. Medical Journal of Qilu, 1997:62-63.
37. Feng DX. Clinical efficacy of licorice root, Salvia miltiorrhiza, and Tripterygium Wilfordii for refractory RAU. The Chinese and foreign health abstract, 2011, 8(47):149-150.
38. Feng L, Ma L. Efficacy and Safety of methotrexate and Tripterygium glycosides tablets for rheumatoid arthritis. Journal of Clinical and Experimental Medicine, 2013, 12(9): 659-661.
39. Feng SZ, Zhang P, Xie L. Treatment of idiopathic membranous nephropathy with small dose of steroid therapy combined with Tripterygium glycosides. Chinese Community Doctors, 2011 (17): 52-52.
40. Fu GK, Lu YH, Li PH. Combination of polysaccharide nucleic acid and Tripterygium Wilfordii for psoriasis vulgaris. Journal of Chinese Medicine Research, 2006, 6(006): 660-661.
41. Fu SX. The Study of the Impact Of serum levels of neuropeptide Y in Psoriasis Vulgaris Patients With TCM Blood-cooling and soothing the liver. Guangzhou University of Traditional Chinese Medicine, 2012.
42. Fu WJ, Ni J, Zheng LJ, et al. Clinical observation of small dose of Tripterygium glycosides for the treatment of hysteromyoma. Journal of Hebei North University, 2005, 22(14): 146-147.
43. Gao DH, Li JH., Tripterygium glycosides combination therapy for purpura nephritis in children. Henan Medical Research, 2000, 9(4): 337-338.
44. Gao F. Low dose of prednisone combined with Tacrolimus and Tripterygium glycosides tablets for idiopathic membranous nephropathy. International Journal of Transplantation and Hemopurification, 2013 (002): 30-33.
45. Gao LJ. Study on clinical related factors and TGP's treatment of Palmoplantar Pustulosis. Tianjin Medical University, 2013.
46. Gao SP. Tripterygium glycosides for the treatment of diabetic nephropathy: a randomized controlled trial. Hainan Medical Journal, 2012, 23(14): 31-32.
47. Gao YP, Chen DP. Clinical study of Tripterygium Wilfordii Hook. F. for hysteromyoma. Chinese Journal of Obstetrics and Gynecology, 2000, 35(7): 430-432.
48. Gao Z, Li GS. Efficacy of Different dosage of Tripterygium glycosides for small vessel vasculitis and proteinuria. Medical Information, 2010, 23(4): 1139-1140.
49. Ge YC. Xie HL, Li SJ, et al. Tripterygium glycosides for the treatment of diabetic nephropathy: a randomized controlled trial. Chinese Journal of Nephrology Dialysis & Transplantation, 2010, 19(6): 501-507.
50. Guan ZX, Chen JH. Tripterygium glycosides combined with the middle dose of prednisone in the treatment of senile primary nephrotic syndrome. Journal of Clinical and Experimental Medicine, 2012, 11(5): 345-346.
51. Guan ZW. The clinical study of children with Henoch-SchÖlein purpura nephritis treated by Qingre Zhixue granules combined with Tripterygium Wilfordii and Danshen injection. Henan University of Traditional Chinese Medicine, 2009.
52. Guo JB, Li ZL, Ma ZY. Etanercept plus Tripterygium wilfordii Hook F for the treatment of rheumatoid arthritis. Medical&Pharmaceutical Journal of Chinese People's Liberation Army, 2011, 8(23): 31-33.
53. Guo LF, Zhang HY, Liu XM. Clinical trial of Qufeng Qingre Huayu decoction for the treatment of 60 patients with rheumatoid arthritis. Hebei Journal of Traditional Chinese Medicine, 2006, 28(9): 660-661.
54. Guo XY, Song YH. Efficacy of Tripterygium glycosides tablets for subacute thyroiditis. Journal of Postgraduates of Medicine, 2001, 24(1): 38-39.
55. Guo XS, Chen XY, Gu YJ. Combination treatment for rheumatoid arthritis. Chinese Journal of Modern Drug Application, 2008, 2(20): 4-5.
56. Guo YQ, Zuo YH. Efficacy of Tripterygium glycosides tablets for diabetic nephropathy. Journal of Clinical Nephrology, 2007, 7(5): 198-199.
57. Guo LF. Clinical study of Qufeng Qingre Huayu decoction for the treatment patients with rheumatoid arthritis. Hebei Medical University, 2007.
58. Han XF, Mou HG, Wang LM. Efficacy of Huoba Huagen tablets for psoriasis. China Journal of Leprosy and Skin Diseases, 2004, 20(6):588-589.
59. He J. Tripterygium glycosides plus acitretin for psoriasis: a controlled trial. Practical Journal of Medicine & Pharmacy, 2008, 25:1188-1189.
60. He XH, Kong DM. Efficacy of Tripterygium glycosides plus the low dose of prednisone for Graves' eye disease. Journal of New Chinese Medicine, 2010 (8): 65-66.
61. He ZS, An ZG, Feng DM, et al. Tripterygium glycosides for the treatment of patients with chronic idiopathic urticarial. The Chinese Journal of Dermatovenereology, 2013, 27(7): 661-664.
62. He ZH. Total glucosides of paeony in the treatment of chronic nephritis proteinuria. Guide of China Medicine, 2011, 9(5): 144-145.
63. Hu K, Wen JY, Mao ZL, et al. Tripterygium glycosides plus mifepristone as add on therapy for after surgery for endometriosis. Chinese Journal of Practical Gynecology and Obstetrics, 2005, 21(7): 438-439.
64. Hu SJ, Cao EZ, Zhang TY, et al. Efficacy of Kangshen Zhixue granules for IgA nephropathy. Journal of New Chinese Medicine, 2005, 37(7): 39-40.
65. Hu SJ, Mao YP, Liu XP, et al. Kangshen Zhixue granules for IgA nephropathy. Clinical Journal of Traditional Chinese Medicine, 2011, 23(3): 234-235.
66. Huang FX. Clinical observation of oral administration of Tripterygium glycosides tablets and Kuiyangling he for the treatment of active ulcerative colitis. Guangzhou University of Traditional Chinese Medicine, 2011.
67. Huang HZ. Clinical observation of Tripterygium glycosides combined with prednisone in the treatment of primary nephrotic syndrome in children. Chinese Primary Health Care, 2012 (4): 90-91.
68. Huang KD. The Clinical Observation and the Study of Mechanism of Xueniaoting Granule Treatment on Henoch-SchÖlein purpura nephritis. Henan University of Traditional Chinese Medicine, 2003.
69. Huang NC. Tripterygium glycosides and irbesartan for diabetic nephropathy. Jilin Medical Journal, 2011, 31(30): 5312-5313.
70. Huang QY. Low Molecular Heparin and Tripterygium glycosides for anaphylactoid purpura nephritis in children. China Foreign Medical Treatment, 2009, 28(7): 72-72.
71. Huang YZ, Ma DX. Isotretinoin and Tripterygium Wilfordii for psoriasis. The Journal of Practical Medicine, 2005, 21(5): 528-529.
72. Huang Y, Long TB, Zhan F, et al. Different Dosage of Tripterygium glycosides for proteinuria after renal transplantation. Journal of Southern Medical University, 2008, 28(12): 2269-2271.
73. Huang ZQ. Clinical trial of tazarotene sotretinoin and Tripterygium glycosides tablets plus for the treatment of 58 cases with psoriasis. Chinese Journal of Experimental Traditional Medical Formulae, 2012, 18(15): 276-278.
74. Huang yz, Huang FR, Ma XD, et al. Clinical observation of Diyin tablet Tripterygium glycosides in the treatment of 52 cases with psoriasis. Chinese Journal of Current Traditional and Western Medicine, 2004, 2(3): 238-239.
75. Ji SG, Wang QW, Yin G, et al. Long term use of Tripterygium glycosides after homotransplantation of the kidney. Journal of Medical Postgraduate, 2007, 20(1): 53-57.
76. Jiang JY, Zhang XW. Clinical efficacy of Feng Tong Ning Pills for the treatment of rheumatoid arthritis. Chinese Journal of Experimental Traditional Medical Formulae, 2009 (9): 97-99.
77. Jiao J, Jiang Q. External application of compound Tripterygium wilfordii decreased the activity of rheumatoid arthritis. Chinese Journal of Integrated Traditional and Western Medicine, 2012, 32(11): 1470-1472.
78. Jie HY,Wu QF. Clinical efficacy of Jinguan tablets for the treatment of 47 patients with rheumatoid arthritis. Clinical Journal of Chinese Medicine, 2013, 4(24): 58-58.
79. Lei T, Zhang XZ, He M, et al. Effect of Multi-glycosides of Tripterygii on Latent Autoimmune Diabetes in Adults in Early Stage. Chinese Journal of Integrated Traditional and Western Medicine, 2006, 6: 009.
80. Li CS, Gao YH, Fan CP, et al. Compare Zhengqing Fengtongning with Tripterygium glycosides for rheumatoid arthritis. Public Medical Forum Magazine, 2005, 9(6): 526-527.
81. Li FL, Cao ZC. Clinical efficacy of Wan Bi Tong for the treatment of 60 patients with rheumatoid arthritis. Modern Journal of Integrated Traditional Chinese and Western Medicine, 2004, 13(6): 739-740.
82. Li J, Yu XG, Ye J, et al. Efficacy of the basic prescription plus syndrome related drugs for the IgA nephropathy. Journal of New Chinese Medicine, 2011, 43(7): 40-42.
83. Li J, Yu XG, Ye J. Clinical study of integrated Chinese and Western medicine for the treatment of refractory nephrotic syndrome. Journal of Chinese Medicine, 2012, 27(9): 1170-1172.
84. Li JH. Tripterygium glycosides tablets plus Huangkui capsule for diabetic nephropathy. Chinese Journal of Primary Medicine and Pharmacy, 2012, 19(18): 12-13.
85. Li RL, Dong SF. Tripterygium sustained release tablets for the treatment of 32 patients with rheumatoid arthritis. Traditional Chinese Drug Research & Clinical Pharmacology, 1995, 6(1): 17-20.
86. Li RL, Liu PL. Clinical and experimental study of Tripterygium sustained release tablets for rheumatoid arthritis. Chinese Journal of Integrated Traditional and Western Medicine, 1996, 16(1): 10-13.
87. Li RL, Wu XC. Tripterygium sustained release tablets verse Tripterygium tablets for rheumatoid arthritis. Chinese Journal of New Drugs and Clinical Remedies, 1995, 14(3): 130-130.
88. Li W, Tang AG, Li AH. Clinical trial of Tripterygium glycosides plus vitamin E for chronic nephritis. Jiangxi Medical Journal, 2009, 44(11): 1107-1108.
89. Li X. Comparison of Tripterygium wilfordii Hook F versus methotrexate in the treatment of rheumatoid arthritis. Peking Union Medical College, 2012.
90. Li RX, Chen MH, Wang HR. Total glucosides of paeony and Tripterygium glycosides in the treatment of 60 patients with rheumatoid arthritis. Journal of Anhui Traditional Chinese Medical College, 2011, 30(3): 16-18.
91. Li YS, Tong PJ, Ma HZ, et al. Toxicity Attenuation and Efficacy Potentiation Effect of Liquorice on Treatment of Rheumatoid Arthritis with Tripterygium Wilfordii. Chinese Journal of Integrated Traditional and Western Medicine, 2006, 26(12): 1117-1117.
92. Li YN, Shi XH. Methimazole combined with Tripterygium Glycosides for the treatment hyperthyroidism. Guide of China Medicine, 2013, 11(14): 133-134.
93. Li ZM, Yang X. Clinical observation of Tripterygium glycosides combined with the small and middle dose of prednisone in the treatment of 30 cases with adult purpura nephritis. Shaanxi Medical Journal, 2010 (11): 1542-1543.
94. Liao JH, Zhao RH, Liu HQ, et al. Short-term efficacy of Yin Dong Zhen Formula for oral lichen planus. The Journal of Practical Medicine, 2009, 25(13): 2181-2182.
95. Lin LM, Qi XM. Comparative observation on the effects of Radix tripterygium hypoglaucum tablet and Tripterygium glycosides tablet in treating erosive oral lichen planus. Chinese Journal of Integrative Medicine, 2005, 11(2):149-150.
96. Lin SP. Tripterygium glycosides plus methotrexate for the treatment of rheumatoid arthritis. Symposium on the 17th National Conference of Chinese Rheumatology, 2012.
97. Liu B, Wang WB. Tripterygium glycosides for the treatment of 30 children with generalized eczema. Beijing Journal of Traditional Chinese Medicine, 1997, 16(1): 27-28.
98. Liu DJ, Liu HH, Wei G, et al. Efficacy of Tripterygium glycosides combined with methylprednisolone for senile nephrotic syndrome. Chinese Journal of Geriatric Care, 2010, 8(5): 51-52.
99. Liu FL. China Tripterygium glycosides and Molecular Heparin for 32 cases of refractory nephrotic syndrome.Modern Medicine, 2009, 16(22): 35-35.
100. Liu GY, Huang Q. Yiqi Qushi Tongluo decoction for the treatment of 360 cases with rheumatoid arthritis. Chinese Journal of Information on Traditional Chinese Medicine, 2005, 12(1): 66-67.
101. Liu JB. Ebastine plus Tripterygium glycosides in the treatment of chronic urticarial. Chinese Community Doctors, 2011 (21): 165-165.
102. Liu JD, Song WL. Tripterygium glycosides for 22 cases with proteinuria after renal transplantation. Chinese Journal of Organ Transplantation, 2005, 26 (7):437-438.
103. Liu Q. Acitretin and Tripterygium glycosides for 35 patients with psoriasis. Chinese Community Doctors, 2006, 8(16): 21-22.
104. Liu QL. Efficacy of Tripterygium glycosides tablets for lupus nephritis. Chinese Journal of Integrated Traditional and Western Nephrology, 2002, 3(10): 609-609.
105. Liu JY, Liu JH, Liu LN, et al. Buxu Quyu treatment in the treatment of rheumatoid arthritis. Hebei Journal of Traditional Chinese Medicine, 2003, 25(5): 332-334.
106. Liu Y. Clinical study of Yanghe decoction for the treatment of rheumatoid arthritis. Fujian University of Traditional Chinese Medicine, 2010.
107. Liu YJ. Tripterygium glycosides plus Acitretin for Palmoplantar Pustulosis. Jinlin University, 2012.
108. Liu ZG. Efficacy of Yishen Qingli Huoxue formula plus Tripterygium glycosides for chronic glomerulonephritis. Journal of Traditional Chinese Medicine, 2013, 28(7): 1385-1386.
109. Liu ZH, Chen JY, Tian ZX, et al. Combined leflunomide with Tripterygium glycosides for refractory nephrotic syndrome. Progress in Modern Biomedicine, 2011, 11(4): 738-741.
110. Luo GL, Chen YJ, Wang J, et al. Efficacy of Xiaoyin decoction for active stage of psoriasis. Modern Chinese Doctor, 2011, 49(2): 43-43.
111. Luo JH, Hu ZH. Efficacy of Tripterygium glycosides for after surgery for endometriosis. Clinical Journal of Medical Officer, 2002, 30(2): 41-42.
112. Luo QJ. Loratadine Dispersible Tablets, Luohua Zizhu tablets and Tripterygium glycosides tablets for acute scrotum eczema. Journal of Medical Front 2013, (21): 110-111.
113. Lv DG. Clinical efficacy of Qubi capsule for the treatment of rheumatoid arthritis., 2001, 24(2): 3-5.
114. Lv Q, Zhang W, Shi Q, et al. Comparison of Tripterygium wilfordii Hook F with methotrexate in the treatment of active rheumatoid arthritis (TRIFRA): a randomised, controlled clinical trial[J]. Annals of the rheumatic diseases, 2014: annrheumdis-2013-204807.
115. Lv XL, Yan MF, Xu MZ. Thalidomide combined with Tripterygium Glycosides in the treatment of Behcet's disease. Journal of Logistics University of CPAPF, 2013 (6): 541-543.
116. Ma Cg. The Clinical Research of Tripterygium glycosides in treating high hypergammaglobulinemia of the patients with primary Sjogren's syndrome. Nanjing University of Chinese Medicine, 2012.
117. Ma HY, HuoYL, Ma XS. Efficacy of acupuncture and herbal medicine for rheumatoid arthritis. Clinical Journal of Traditional Chinese Medicine, 2008, 20(3): 291-293.
118. Meng B, Gao LZ, Zhao HP. Clinical trial of compound Tripterygium wilfordii wine for rheumatoid arthritis. Information on Traditional Chinese Medicine, 2013 (1): 94-95.
119. Meng FJ. Ornidazole plus Tripterygium glycosides for acne. The Chinese Journal of Dermatovenereology, 2005, 19(4): 214.
120. Meng P. Efficacy of Tripterygium glycosides for Graves' eye disease. Guiyang College of Traditional Chinese Medicine, 2011.
121. Meng QL, Guo HQ, Zhao YJ. Efficacy of Yishen Juanbi decoction and methotrexate for 40 patients with rheumatoid arthritis. Traditional Chinese Medicinal Research, 2006, 19(8): 27-29.
122. Mo XX. Combined leflunomide with Tripterygium glycosides for refractory nephrotic syndrome. Chinese Community Doctors, 2009 (10): 54-55.
123. Mou T. Chinese herbal decoction and external application for rheumatoid arthritis. Journal of Chinese Medicine, 2013, 28(9): 1386-1387.
124. Nie YH. Effcicy of different combination treatment for nephrotic syndrome. Chinese Journal of Modern Drug Application, 2010, 4(22): 96-97.
125. Pang FM. Clinical trial of Tripterygium glycosides for purpura nephritis. Hainan Medical Journal, 2011, 22(10): 84-85.
126. Qi AR, Li SM, Xiong GL, et al. Tripterygium glycosides plus prednisone for IgA nephropathy: 20 cases trial. Chinese Journal of Traditional Medical Science and Technology, 2011, 22(10): 84-85.
127. Qi YQ, Wang HP, Hou SP, et al. Compound licorice in the treatment of patients with psoriasis vulgaris. The Chinese Journal of Dermatovenereology, 2009, 23(2): I0001-I0002.
128. Qian JQ, He YX. . Tripterygium glycosides for IgA nephropathy. Chinese Journal of Nephrology, 1989, 5(1): 21-25.
129. Qian YY, Li YT. Short term use of Tripterygium glycosides after homotransplantation of the kidney. Chinese Journal of Organ Transplantation, 1993, 14(3): 133-135.
130. Qu QW. Wudang Chinese medicine steaming treatment for 100 cases with rheumatoid arthritis. Traditional Chinese Medicinal Research, 2002, 15(3): 31-33.
131. Qu SZ. Clinical observation and metabolic phenotypic characteristics on psoriasis vulgaris treated by Yin Xie. Guangzhou University of Traditional Chinese Medicine, 2012.
132. Goldbach-Mansky R, Wilson M, Fleischmann R, et al. Comparison of Tripterygium wilfordii Hook F versus sulfasalazine in the treatment of rheumatoid arthritis: a randomized trial[J]. Annals of internal medicine, 2009, 151(4): 229-240.
133. Ren J, Wu X, Liao N, et al. Prevention of postoperative recurrence of Crohn’s disease: Tripterygium wilfordii polyglycoside versus mesalazine[J]. Journal of International Medical Research, 2013: 0300060512474744.
134. Ren JX. Erythromycin, Triptolide and scaly polypeptide in the treatment of 66 patients with psoriasis. Zhonghua Yixue Xiezuo Zazhi, 2000, 7(2): 213-214.
135. Ruan XY, Shao KW, Zhang JT. Efficacy of Tripterygium Wilfordii Hook. F. for neurodermatitis disseminate. Fujian Journal of Traditional Chinese Medicine, 1988, 5: 029.
136. Shao JH. Clinical study of integrated Chinese and Western medicine for the treatment of 33 cases of rheumatoid arthritis. Zhejiang Journal of Integrated Traditional Chinese and Western Medicine, 2014, 24(1): 46-49.
137. Shen J, Zhang ZL. Tripterygium glycosides plus low dose of methotrexate for the treatment of elderly rheumatoid arthritis. Zhejiang Journal of Integrated Traditional Chinese and Western Medicine, 2002, 12(6):334-336.
138. Shen LR, Zhang LL, Chi BX, et al. Efficacy of double dose of Tripterygium glycosides for IgA nephropathy. Youjiang Medical Journal, 2011, 39(2): 142-144.
139. Shen RZ, Qiu RB, Deng HW. Qingfengtang decoction for 45 cases of rheumatoid arthritis. Journal of New Chinese Medicine, 2004, 36(9): 26-27.
140. Sheng YZ, Yu XB, He Y, et al. Clinical trial of Tripterygium wilfordii mixture for type 2 leprosy. China Journal of Leprosy and Skin Diseases, 2012, 28(5): 365-366.
141. Sheng MX, Sun W, Xing CY, et al. Treatment of Chronic Primary Glomerulopathy Patients of Shen Deficiency and Dampness Heat Syndrome by Yishen Qingli Granule Combined Low-dose Tripterygium Wilfordii Multiglycoside Tablet: a Clinical Efficacy Observation. Chinese Journal of Integrated Traditional and Western Medicine, 2013, 33(12): 1640-1640.
142. Shi TY, Zhao XJ. Tripterygium glycosides plus Jinshuibao capsule in treatment of 76 diabetic nephropathy patients with proteinuria. China's rural health, 2013 (03Z): 203-204.
143. Shi YJ, Liu ZY, Wang C, et al. Tripterygium glycosides for early stage diabetic nephropathy. Modern Journal of Integrated Traditional Chinese and Western Medicine, 2006, 15(8): 987-988.
144. Song B. Compare cyclophosphamide with Tripterygium glycosides for refractory nephrotic syndrome in Children. China Practical Medicine, 2008, 3(14): 7-8.
145. Song X. Tripterygium glycosides combined with vincristine for ITP. Journal of Qiqihar Medical College, 2012, 33(13): 1744-1745.
146. Song YN. Clinical study of Tripterygium glycosides tablets for ankylosing spondylitis. Nanjing University Of Chinese Medicine, 2010.
147. Su CH, Hao RH. Tripterygium glycosides plus triamcinolone acetonide injectable for acne. Journal of Dermatology and Venereology, 2010 (3): 37-37.
148. Sun DH, Shi Q. Clinical study of two DMARDs combination therapy for rheumatoid arthritis. Chinese Remedies & Clinics, 2003, 3(3): 203-204.
149. Sun JP, Gao YX, Dong H. Sinomenine preparation for treating 28 patients with chronic glomerulonephritis. Clinical Focus, 2007, 22(16): 1194-1195.
150. Kong RL. Yinxieping decoction for psoriasis vulgaris: a 110 cases trial. Jiangsu Journal of Traditional Chinese Medicine, 2004, 8: 015.
151. Tang GQ, Gu Y, Zhang HY. Efficacy of mifepristone plus Tripterygium glycosides for hysteromyoma. China Foreign Medical Treatment, 2009 (25): 80-81.
152. Tang XL, Hong SJ, Tang LL. Efficacy of Tripterygium glycosides and Ticlopidine for refractory nephrotic syndrome. Chinese Journal of Integrated Traditional and Western Nephrology, 2004, 5(1): 27-27.
153. Tang XS. Ornidazole, Tripterygium glycosides and Electrocauterization for acne conglobate. The Chinese Journal of Dermatovenereology, 2007, 21(6): 347-348.
154. Tang YP. Chinese herbal medicine Zoumatai for rheumatoid arthritis. Journal of Sichuan of Traditional Chinese Medicine, 2007, 25(1): 54-55.
155. Tao K. Combined Tripterygium glycosides with prednisone for adult purpura nephritis. The Chinese and foreign health abstract, 2010, 7(36).
156. Tao X, Younger J, Fan F Z, et al. Benefit of an extract of Tripterygium Wilfordii Hook F in patients with rheumatoid arthritis: A double‐blind, placebo‐controlled study[J]. Arthritis & Rheumatism, 2002, 46(7): 1735-1743.
157. Tian XM, Wang ZM. Traditional formule for rheumatoid arthritis: a 24 cases trial. Traditional Chinese Medicinal Research, 2009, 22(9): 23-24.
158. Tian Y. Efficacy of Levocetirizine Dihydrochloride plus Tripterygium glycosides for chronic urticarial. Guangming Journal of Traditional Chinese Medicine, 2012, 27(1): 105-106.
159. Quan Y, Ji YL, Li YY, et al. Clinical observation of Tripterygium glycosides for the treatment of III diabetic nephropathy. 2012, 7(011): 1418-1420.
160. Tu SH, Hu YH. Tripterygium Wilfordii Hook F for patients with rheumatoid arthritis: efficacy, and quality of life. Journal of Hunan University of Traditional Chinese Medicine, 2006, 26(2):25-27.
161. Wan SF, Liu GM. Combined leflunomide with Tripterygium glycosides for severe rheumatoid arthritis. West China Medical Journal, 2009, 24(6): 1461-1463.
162. Wang B, Huang ZF. Clinical observation of quadruple therapy in treatment of prurigo Nodularis. Practical Clinical Journal of Integrated Traditional Chinese and Western Medicine, 2013, 13(4): 43-44.
163. Wang D. Efficacy of Ebastine Combined with Tripterygium Glycosides in the treatment of chronic urticarial. Medical Information, 2010, 23(22): 4215-4217.
164. Wang HJ. Clinical study of Yishen Qingli Huoxue formula plus Tripterygium glycosides for chronic glomerulonephritis. Chinese Medicine Guides, 2012, 9(27): 88-89.
165. Wang HW, Chen JH. Tripterygium glycosides combined with prednisone in the treatment of senile nephrotic syndrome. Journal of Youjiang Medical College for Nationalities, 2012, 9(27): 88-89.
166. Wan J, Wang ZL, Du H, et al. Tripterygium wilfordii polyglycoside and corticosteroid in the treatment of severe hyperthyroidism, Journal of Medical Postgraduate, 1999, 12(4): 218-218.
167. Wang JM. Wang J, Yang L, et al, Tripterygium glycosides combined with Losartan for the treatment of stage IV diabetic nephropathy. Chinese Journal of Practical Medicine, 2013, 39(22): 123-124.
168. Wang J. Efficacy of homemade Tripterygium Plaster for Pain from Ankylosing spondylitis. Zhejiang Chinese Medical University, 2013.
169. Wang JH, Liu J. Tripterygium glycosides for anaphylactoid purpura nephritis. Journal of Chinese Medicine, 2011, 26(4): 490-491.
170. Wang LJ, Liu D, Shen L. Efficacy of homemade Huangteng wine for rheumatoid arthritis. Practical Journal of Medicine & Pharmacy, 2012, 29(7): 614-615.
171. Wang ML, Zhang C. Tripterygium glycosides tablets plus Valsartan for patients with renal proteinuria. Linchuang Heli Yongyao Zazhi, 2012, 5(22): 84-85.
172. Wang M. Combination therapy for lupus nephritis: a 52 cases trial. Henan Medical Information, 1999, 7(11): 11-12.
173. Wang Y, Wu Q, Wei JX, et al. Total glucosides of paeony, methotrexate and Tripterygium glycosides in the treatment of 150 patients with rheumatoid arthritis. Journal of Zhengzhou University (Medical Sciences), 2006, 41(5): 1002-1003.
174. Wang W. Yure Zheng in the treatment of rheumatoid arthritis. Collection of the 6^th^ International Congress of Acupuncture and Traditional Medicine, 2000.
175. Wang YL. Clinical study of integrated Chinese and Western medicine for the treatment of refractory nephrotic syndrome. Central Plains Medical Journal, 2006, 33(10): 60-61.
176. Wang YH, Gao YL. Tripterygium glycosides in the treatment of patients with chronic renal insufficiency. Jiangxi Journal of Traditional Chinese Medicine, 2003, 34(1): 20-21.
177. Wang YJ, Zhang LM, Men GG. Tripterygium glycosides plus prednisone for purpura nephritis in children. Journal of Pediatric Pharmacy, 2012, 18(6): 24-24.
178. Wang YL, Zhang LM, Xin RZ. Tripterygium glycosides plus prednisone for naphylactoid purpura nephritis in children: 56 cases trial. Chinese Journal of Coal Industry Medicine, 2007, 10(2): 161-162.
179. Wang ZQ. Clinical and experimental study of Tripterygium Wilfordii Hook. F. liposomes chitosan for the treatment of active rheumatoid arthritis. Guangzhou University of Traditional Chinese Medicine, 1999.
180. Wei W. Clinical and experimental study of Tripterygium Wilfordii Hook. F. external preparation for psoriasis vulgaris. The 4^th^ National meeting of Tripterygium Wilfordii Hook. F. 2004.
181. Wen Q. Efficacy of Tripterygium glycosides plus mifepristone for hysteromyoma. Clinical Medicine of China, 2005, 21(3):277-279.
182. Wen XS. Efficacy of low dosage of Tripterygium glycosides for the treatment of early rheumatoid arthritis. Wei Wu Er Yi Yao, 2013, (4):245-247.
183. Wu CZ. Double dosage of Tripterygium glycosides plus Telmisartan for the treatment of 32 patients with early diabetic nephropathy. Herald of Medicine, 2010, 29(9):1173-1175.
184. Wu GX. Combined Tripterygium glycosides with prednisone for adult purpura nephritis. Journal of Medical Forum, 2012, (6):52-53.
185. Wu HJ, Liu CG, Liu JY. Tripterygium glycosides tablets for the treatment of diabetic nephropathy. Journal of Medical Forum, 2011, 23(4):389-391.
186. Wu SB, Cao ZD, Zhang W, et al. Tripterygium glycosides plus Olmesartan medoxomil for the treatment of early diabetic nephropathy. 2011, 11(12):1388-1390.
187. Wu W. Tripterygium glycosides for postmenopausal bleeding recurs in a menopausal woman. Sichuan medicine, 2012, 33(6):1015-1017.
188. Wu XM, Kang EZ, Yu YS, et al. No 82 Tripterygium wilfordii syrup in the treatment of 106 patients with rheumatoid arthritis. Pharmacology and Clinics of Chinese Materia Medica, 1988, 4(3): 50-52.
189. Wu YJ, Lao ZY, Zhang ZL. Clinical observation on small doses Tripterygium wilfordii polyglycoside combined with methotrexate in treating rheumatoid arthritis. Chinese Journal of Integrated Traditional and Western Medicine, 2001, 21(12):895-896.
190. Xiao HQ, Zhang JE, Ye CY, et al. Efficacy of Tripterygium glycosides for senile primary nephrotic syndrome. Journal of Clinical Nephrology, 2001, (3):131-133.
191. Xiao HQ, Ding GH, Zhang JE, et al. Tripterygium glycosides combined with cyclophosphamide in the treatment of senile primary nephrotic syndrome. Clinical Focus, 2003, 18(18):1039-1040.
192. Xiao YC. Tripterygium glycosides in the treatment of 50 patients with senile primary nephrotic syndrome. Chinese Journal of General Practice, 2007, 5(4):312-313.
193. Xie T, Zhong YX, Mai WM, et al. Clinical trial of Tripterygium glycosides for IgA nephropathy. Chinese Journal of Postgraduates of Medicine, 2008, 31(1):35-36.
194. Xie T. Treatment of chronic kidney disease with Tripterygium glycosides therapy. Modern Journal of Integrated Traditional Chinese and Western Medicine, 2007, 16(26):3787-3789.
195. Xin LN, Zhang LL. Tripterygium wilfordii polyglycoside and corticosteroid in the treatment of IgA nephropathy: a controlled trial．Journal of Clinical Medicine in Practice, 2011, 15(21):103-104.
196. Xin XF, Chen Y. Clinical study of integrated Chinese and Western medicine for the treatment of elderly rheumatoid arthritis. Zhejiang Journal of Integrated Traditional Chinese and Western Medicine, 2005, 15(5):292-293.
197. Xing F, Lin WY, Xu XW, et al. Tripterygium wilfordii decoction, methotrexate and sulfasalazine for ankylosing spondylitis. China medicine, 2006, 1(2):75-76.
198. Xu F, Dai XM, Zhang GH, et al. Siteng decoction for 123 cases with rheumatoid arthritis. Journal of Traditional Chinese Medicine, 2003, 44(10):760-761.
199. Xu PP. Efficacy of Tripterygium glycosides and mifepristone for endometriosis. Chinese Journal of Misdiagnostics, 2011, 11(31):7618-7618.
200. Xu W. Clinical study of Tripterygium glycosides tablets for rheumatoid arthritis. Fujian college of traditional medicine, 2000.
201. Xu XH, Hu SY. Tripterygium glycosides tablets for 20 patients with subacute thyroiditis. Clinical education of general practice. 2011, 09(3):342-343.
202. Xue S. Tripterygium wilfordii polyglycoside and corticosteroid in the treatment of refractory nephrotic syndrome. Hubei University of Traditional Chinese Medicine, 2012.
203. Yang DW. Qingteng decoction for 52 cases of rheumatoid arthritis patients. International Conference & Exposition on Traditional medicine, 2009.
204. Yang F, Wang JL, An XH, et al. Huangteng wine for 159 cases of rheumatoid arthritis patients. Modern Journal of Integrated Traditional Chinese and Western Medicine, 2002, 11(24):2448-2449.
205. Yang FY, Wei CY, Li CY. Tripterygium glycosides for IgA nephropathy: a controlled trial. Chinese Journal of General Practice, 2008, 6(11):1138-1139.
206. Yang JL, Zhang LZ. Efficacy of TwHF for Acute nephritis in children. Journal of Clinical Pediatrics, 1987, 5(3):162-163.
207. Yang M, Zhou RH, Li BZ, et al. Combined methotrexate with Tripterygium glycosides for the treatment of rheumatoid arthritis. Chinese Journal of Experimental Traditional Medical Formulae, 2013, 19(17):300-304.
208. Yang WY, Zhao XL, Cui XL, et al. Clinical study of Jiawei Simiao capsule rheumatoid arthritis. Journal of Traditional Chinese Medicine, 2004, 19(5):40-42.
209. Yang XY, Zhang L. Tripterygium glycosides tablets for the treatment of 60 patients with rheumatoid arthritis. Chinese Journal of Traditional Medical Science and Technology, 2007, 14(2):130-131.
210. Yang Y, Wang CL, Lin X, et al. Tripterygium glycosides plus Chinese herbal medicine for Henoch-Schönlein Purpura Nephritis in Children. Journal of Emergency in Traditional Chinese Medicine, 2008, 17(11):1535-1536.
211. Yang Z. Tripterygium tablets for the treatment of 74 patients with rheumatoid arthritis. China Pharmaceuticals, 2011, 20(14):76-77.
212. Yao H, Sun XD, Yao J, et al. Clinical study Quhan Chushi Huoxue method for rheumatoid arthritis. Chinese Journal of Basic Medicine in Traditional Chinese Medicine, 2010, (7):584-585.
213. Yao WC, Nian HF. Tripterygium wine for 56 patients with severe rheumatoid arthritis. The 4th National meeting of Tripterygium Wilfordii Hook. F.,2004.
214. Hua YM, Chen WL, Shen H. Clinica efficacy of high dose of Tripterygium glycosides for chronic nephritis proteinuria.Contemporary Medicine, 2008, (5): 140-142.
215. Yin C, Liang M, Wang X. Efficacy of triamcinolone Acetonide and/ or Tripterygium for oral lichen planus. Journal of Dental Prevention and Treatment, 1996, (1):18-19.
216. Yu M, Li LH, Yu Z, et al. Yishen decoction plus Huoba Huagen tablets for 42 cases with IgA nephropathy. Information on Traditional Chinese Medicine, 2006, 23(6):24-25.
217. Yu Y, Chen HJ. Efficacy of Jianpi Bushen Qufeng method for severe rheumatoid arthritis. Journal of Guangdong medical college, 2005, 23(4):410-412.
218. Yuan DS, Wang YR. The middle dose of prednisone combined with Tripterygium in the treatment of senile primary nephrotic syndrome. Journal of China clinical medicine, 2003, 5(6):11475-11476.
219. Yuan XY. Dosage and adverse effect of Tripterygium glycosides. Chinese Journal of Clinical Rehabilitation, 2002, 6(12):1808-1808.
220. Yuan ZW, Yuan YX. Clinical study of Futeng decoction for 52 cases of rheumatoid arthritis. The 4^th^ National meeting of Tripterygium Wilfordii Hook. F. 2004.
221. Yuan L. The observation of clinical effect of adverse drug reaction on Children patients of HBV-GN by using different dosage of Tripterygium combined with TCM of Ganshenbao Prescription. Henan University of Traditional Chinese Medicine, 2007.
222. Yue ZF, Han XS, Liu XD, et al. Compound licorice plus Tripterygium glycosides in the treatment of patients with eczema. Journal of Guiyang Medical College, 2013, 38(5):541-542.
223. Zeng J, Wang J, Gao XQ. Chuanteng decoction for 82 cases of rheumatoid arthritis. Journal of Sichuan of Traditional Chinese Medicine, 2004, 22(6):34-36.
224. Zeng LL. Tripterygium glycosides plus prednisone for purpura nephritis in children. Journal of Liaoning University of Traditional Chinese Medicine, 2013, (6):182-183.
225. Zhang AP, Zhang L, Wang YX, et al. Clinical study of leflunomide for anaphylactoid purpura nephritis. Chinese Remedies & Clinics, 2004, 4(5):350-352.
226. Zhang DP, Lai HL. Roxithromycin plus Tripterygium glycosides in the treatment of severe acne. Nei Mongol Journal of Traditional Chinese Medicine, 2011, 30(11):60-61.
227. Zhang F. Prednisone monotherapy verse prednisone plus Tripterygium glycosides in the treatment of primary nephrotic syndrome. The Chinese and foreign health abstract, 2007, (8):96-98.
228. Zhang G, Fu ZL, Wang K, et al. Zhengqing Fengtongning for Rheumatoid arthritis. Proceeding of Clinical Medicine, 2007, 14:893-895.
229. Zhang GL. Clinical efficacy of Tripterygium glycosides for the treatment of diabetic nephropathy. Neifenmi Daixiebing Zhongxiyijiehe Yanjiu, 2010.
230. Zhang HY, Wang ZF. Clinical observation of Tripterygium glycosides for the treatment of pityriasis rosea. Chinese Traditional Patent Medicine, 1998, (11):24-24.
231. Zhang H. Tripterygium glycosides tablets plus Valsartan for patients with early diabetic nephropathy. Chinese Journal of Modern Drug Application, 2009, 3(14):94-95.
232. Zhang HQ. Clinical efficacy of Mifepristone combined with Tripterygium glycosides for 38 women with hysteromyoma. Modern Chinese Doctor, 2011, 49(24):97-98.
233. Zhang HR. Efficacy of Tripterygium glycosides plus Mycophenolate MofetilChinese for senile IgA nephropathy. Medicine Guides, 2013, 10(1):81-82.
234. Zhang HY, Yan HZ, Xie KH. Efficacy of Mifepristone combined with Tripterygium glycosides for hysteromyoma. Modern Journal of Integrated Traditional Chinese and Western Medicine, 2010, 19(2):168-169.
235. Zhang QL. Shenyanling granule verse Tripterygium preparations for chronic glomerulonephritis. Guide of China Medicine, 2013, (29):478-479.
236. Zhang QL. Qingdai capsule and Tripterygium glycosides for the treatment of 38 cases with pityriasis rosea. Journal of Dermatology and Venereology, 2007, 29(1):23-24.
237. Zhang Q. Professor Zhou Naiyu’ clinical experience and clinical study of No I Biyukang decoction for advanced RA (Hanshi Bizu Syndrome). Beijing University of traditional Chinese medicine, 2011.
238. Zhang QL. Efficacy of Tripterygium glycosides plus desloratadine for chronic urticarial. Modern Journal of Integrated Traditional Chinese and Western Medicine, 2013, 22(12):1316-1318.
239. Zhang X, He XM, Du WB, et al. Clinical observation of Tripterygium glycosides combined with the small and middle dose of prednisone in the treatment of senile primary nephrotic syndrome. Chinese Journal of Primary Medicine and Pharmacy, 2010, 17(5):623-625.
240. Zhang X, Bao H, Tang Z, et al. Clinical efficacy of Triple therapy for IgA nephropathy. Chinese Journal of Nephrology Dialysis & Transplantation, 2007, 16(3):209-214.
241. Zhang XC, Shi FQ, Ji HW. Pan Long Qi tablets for 60 cases with rheumatoid arthritis: clinical study. Modern Traditional Chinese Medicine, 2011, 31(6):41-42.
242. Zhao HP, Yang DW, Wang SM, et al. Efficacy of Wubang Wan for Rheumatoid arthritis. Liaoning Journal of Traditional Chinese Medicine, 2006, 33(2):189-190.
243. Zhao HY. Combined leflunomide with prednisone for anaphylactoid purpura nephritis: a randomized controlled trial. Chinese Journal of Modern Drug Application, 2011, 05(23):1-3.
244. Zheng BL, Yu JW, Zhang XJ, et al. No 2 Tongbi decoctions for the treatment of 80 patients with rheumatoid arthritis. Shandong Journal of Traditional Chinese Medicine, 2010, (8):528-529.
245. Wang LN, Tang XZ, Chen YP. Tripterygium glycosides combined with prednisone in the treatment of nephrotic syndrome. International meting 2011 Nephrology in Integrated Traditional and Western Medicine, 2001.
246. Zhong H, Hou XL, Xu XZ. Clinical trial of Tripterygium glycosides in the treatment of lipid disorders in nephrotic syndrome. Chinese Journal of Traditional Medical Science and Technology, 2012, 19(2): 156-157.
247. Zhou P. Efficacy of Tripterygium preparations plus Propylthiouracil in the treatment of toxic diffuse goiter. Journal of Zhejiang Chinese Medical University, 2008, 5(6): 406-408.
248. Zhou SL. Efficacy of low dose of Tripterygium glycosides for idiopathic membranous nephropathy. Chinese Journal of Integrated Traditional and Western Nephrology, 2010 (8): 718-719.
249. Zhou TG, Deng ZH, Huang SW. The effect of Tripterygium glycosides for glucocorticoid receptor in Children with Henoch-Schönlein Purpura Nephritis. Research, 2007, 2: 0.01.
250. Zhou XL, Bai W, Chen XD. Clinical study of Tongluo Zhixue granules for rheumatoid arthritis patients with Chinese medicine damp and hot syndrome. Zhongguo Xin Yiyao, 2004, 3(5): 68-70.
251. Zhou XH, Yao CY. Clinical trial of Tripterygium glycosides for 30 cases of IgA nephropathy. Modern Chinese Doctor, 2010 (30): 34-35.
252. Zhou XP, Zhou ZY. Clinical study of Qingre Tongbi granules for rheumatoid arthritis. Journal of traditional Chinese medicine, 2003, 44(3): 191-193.
253. Zhou XP, Liu ZT. Clinical study of Qingluo Tongbi decoction for rheumatoid arthritis. Forum on Traditional Chinese Medicine, 2004, 19(2):23-25.
254. Zhou Y, Fan XZ, Yao LC, et al. Efficacy of Tripterygium tablets for the treatment of 94 cases of alopecia areata. Journal of New Medicine, 2000, 31(3):162-163.
255. Zhou YC. Clinical observation of Tripterygium glycosides combined with the low dose of prednisone in the treatment of nephrotic syndrome. Hebei Medicine, 1999, (4):14-16.
256. Zhou YZ. Clinical study of integrated Chinese and Western medicine for the treatment of primary nephrotic syndrome. Hebei Journal of Traditional Chinese Medicine, 2000, 22(8): 620-621.
257. Zhou ZK, Ke Q. Clinical efficacy of combined NB-UVB with Tripterygium preparations for psoriasis. Hainan Medical Journal, 2012, 23(2): 82-83.
258. Zhou ZS, Yuan ZW. Clinical study of Tripterygium and Radix Angelicae Sinensis wine for rheumatoid arthritis. Branch committee of rheumatology of China association of Chinese medicine 2010.
259. Zhu FX, Fang RH, Shi YH. Methotrexate plus Hydroxychloroquine or Tripterygium glycosides for the treatment of early rheumatoid arthritis. Guide of China Medicine, 2013, 10(18): 416-418.
260. Zhu H, Ge ZM, Liu YL, et al. Tripterygium preparations for systemic lupus erythematosus: a randomized, double-blind trial. Journal of Taishan Medical College, 1998, 4: 027.
261. Zhu KJ, Chen LC. Clinical trial of Zhengqing Fengtongning tablets in the treatment of chronic nephritis proteinuria. Chinese Journal of New Drugs and Clinical Remedies, 1999, 10(2): 67-70.
262. Zhu L, Chen P, Zhou R, et al. Efficacy of Tripterygium glycosides, glucosamine sulfate and potassium diacerein for osteoarthritis. Acta Academiae Medicinae Xuzhou, 2013, 33(006): 391-394.
263. Zou RY. Peony & Anemarrhena Decoction with Additions for the treatment of 30 patients with rheumatoid arthritis. Journal of Emergency in Traditional Chinese Medicine, 2007, 16(6): 672-673.
264. Zuo YM. Clinical study of Qubi herbal remedy for rheumatoid arthritis. The Chinese and foreign health abstract, 2008, 5(6): 406-408.

**Controlled clinical trials**

1. Ao JH, Qian YY. Clinical study of Tripterygium glycosides for patients after homotransplantation of the kidney. Chinese Journal of Surgery, 1994, 32(3): 175-177.
2. Cao NL, Zhang XY, Xu YH, et al. Efficacy and safety of different dosage of Tripterygium glycosides and Molecular Heparin for purpura nephritis. Chinese Pediatrics of Integrated Traditional and Western Medicine, 2013, 5(3): 223-224.
3. Qin WZ. Low dose of prednisone plus methotrexate and Tripterygium glycosides for rheumatoid arthritis. Medical Information, 2013:256.
4. Chen LN, Yu DY, Li H. Tripterygium Wilfordii Hook. F. leaf preparation for rheumatoid arthritis: a 32 cases clinical study. Journal of Medical Postgraduate, 1991 (2): 105-107.
5. Chen Q, Zhu YY, Han WL, et al. Zhixue Huoxue formula verse Tripterygium glycosides for renal hematuria: a controlled study. Chinese Journal of Integrated Traditional and Western Nephrology, 2012, 13(1):70-71.
6. Cui Y, Dai XL. Efficacy of Tripterygium glycosides for patients with uveitis. Journal of Changzhi Medical College, 2001, 15(3): 212-213.
7. Dai XN, Wang LX, Xu LH, et al. Clinical efficacy of combined NB-UVB with Tripterygium tablets for 156 patients with hand eczema. The Chinese Journal of Dermatovenereology, 2009, 16(04): 224-227.
8. Ding YM. Low dose of Tripterygium preparation for rheumatoid arthritis. Journal of Gansu College of Traditional Chinese Medicine, 1995, 3: 12-14.
9. Duan XF, Liu ZH. Clinical study of Yubi pills for rheumatoid arthritis. Chinese Journal of Traditional Medical Traumatology & Orthopedics, 1998, 6(6): 21-24.
10. Feng GQ, Song Y, Li YM. Clinical observation of Chinese herbal medicine in the treatment of 30 patients with photosensitivity diseases. The national Chinese medicine dermatology Conference, 2002.
11. Fu P, Gong Y. Sex hormone levels of women with Tripterygium preparation induced amenorrhea. Journal of Clinical Dermatology, 1999, 28(3): 163-164.
12. Gao CJ, Chen XP. Mineral bath therapy verse Tripterygium glycosides for patients with rheumatoid arthritis. Chinese journal of physical therapy, 1995, 18(4): 228-229.
13. Gao JJ, Ji N. Sinomenine preparation monotherapy verse combined with Hydroxychloroquine or Tripterygium glycosides: a controlled trial. Chinese Journal of New Drugs and Clinical Remedies, 1999, 18(4): 215-217.
14. Gao YJ. Tripterygium preparation plus Wudai ointments for the treatment of generalized eczema. Practical Journal of Clinical Medicine, 2011, 8(6): 162-163.
15. Song ZQ. Clinical observation using of Tripterygium wifordii Unites Chai Ling Tang to treat the chronic renal Nephritis dampness-Heat symptom. Nanjing University of Chinese Medicine, 2008.
16. Gong R. Clinical study of integrated Chinese and Western medicine for the treatment of nephrotic syndrome. Journal of Sichuan of Traditional Chinese Medicine, 2007, 25(2): 70-71.
17. Guo QY. Tripterygium glycosides, benazepril and Molecular Heparin for purpura nephritis in children. Chinese Journal of Primary Medicine and Pharmacy, 2010 (9): 1241-1242.
18. Han ZM, Zhang H, Zhao DA, et al. Clinical efficacy of leflunomide for anaphylactoid purpura nephritis. China medicine, 2006, 1(11): 690-691.
19. He QB, Yin WG, Yu JB. Efficacy of Acitretin verse Tripterygium glycosides for patients with psoriasis. China Journal of Leprosy and Skin Diseases, 2005, 20(4): 342-343.
20. He SH, Dai CK, Hu HB. Efficacy of steroid therapy, Tripterygium glycosides for lupus nephritis. Acta Academiae Medicinae Wannan, 1993, 12(3): 211-212.
21. Hu J, Li CW. Efficacy of sequential therapy for children with systemic lupus erythematosus. Chinese Journal of Practical Pediatrics, 2001, 16(10): 612-614.
22. Dermatology of Chinese Academy of Medical Sciences. Tripterygium wilfordii Hook F in the treatment of lepriasis. Acta Academiae Medicinae Sinicae, 1979, (1):71-75.
23. Ji HW, Yao SL. Clinical trial of Bushen Huoxue method for 43 cases of rheumatoid arthritis. Journal of Traditional Chinese Medicine, 2001, 42(10): 609-610.
24. Ji S M, Wang Q W, Chen J S, et al. Clinical trial of Tripterygium wilfordii Hook F. in human kidney transplantation in China[C]//Transplantation proceedings. Elsevier, 2006, 38(5): 1274-1279.
25. Ji S M, Li L S, Wen J Q, et al. Therapeutic effect of Tripterygium wilfordii on proteinuria associated with sirolimus in renal transplant recipients[C]//Transplantation proceedings. Elsevier, 2008, 40(10): 3474-3478.
26. Jiang W, Li LS, Tang Z, et al. Clinical study of integrated Chinese and Western medicine for the treatment of refractory nephrotic syndrome. Journal of Medical Postgraduate, 1988, 2: 001.
27. Jiangsu Group of Tripterygium research. Clinical efficacy of Tripterygium wilfordii for leprosy. Journal of Chinese Medicine Research, 1976, (4): 23-28.
28. Lao ZY. Adverse effects of Tripterygium glycosides for rheumatoid arthritis. Chinese Journal of New Drugs and Clinical Remedies, 1988, 7(1): 51-52.
29. Li JX, Zhu SQ. Clinical study of Tripterygium topical agent for leucoderma in children. Southern China Journal of Dermato-Venereology, 1995, 2(4): 34-34.
30. Li QH, Hu ZX, Huang MG. A clinical observation on the correlation of dose and therapeutic when treating the proteinuria of Chronic glomerulonephritis with GTW. Modern Journal of Integrated Traditional Chinese and Western Medicine, 2005, 14(11): 1429-1430.
31. Li RL. Tripterygium pills for rheumatoid arthritis: a pilot study. Pharmacology and Clinics of Chinese Materia Medica, 1988, 2: 016.
32. Li XL. Tripterygium glycosides and proteinuria amount. University Of Chinese Medicine, 2010.
33. Li XY, Fang WJ. Efficacy of Huoba Huagen tablets for 60 patients with IgA nephropathy Chinese Herbal Medicines, 2001, 32(9):825-826.
34. Li YL, Chen QH. The Effect of Intravenous Cyclophospamide Therapy on Henoch schoenlein Purpura Nephritis of Children. Suzhou University Journal of Medical Science, 2005, 25(2): 314-316.
35. Li YY, Zhong WJ, Chi LQ. Acitretin plus Tripterygium glycosides for Palmoplantar Pustulosis. Chinese Community Doctors, 2010 (19): 16-16.
36. Li ZH. Qiteng Huoluo tablets and Tripterygium glycosides tablets for rheumatoid arthritis: a controlled trial. Pharmaceutical research, 23-25.
37. Lin LM, Qi XM. Huoba Huagen tablets plus Tripterygium glycosides tablets for oral lichen planus. Chinese Journal of Integrated Traditional and Western Medicine, 2004, 5: 029.
38. Lin Z. Clinical effect on tubulointerstitial injury of Ig A nephropathy with Shen Yan Zhi Xue Poul. Heilongjiang Chinese Medicine Research Institute, 2008.
39. Liu JS, Wang XB, Zhao HM. Tripterygium glycosides plus dipyridamole and valsartan for chronic nephritis. Journal of Clinical Nephrology, 2005, 4(6): 279-281.
40. Liu JH, Zhang XW, Yin XF, et al. Clinical efficacy of Qufeng Huoluo pills for rheumatoid arthritis. China Pharmacist, 2006, 9(5): 425-427.
41. Liu XQ, Zeng HM. Tripterygium combined with prednisone in the treatment of anaphylactoid purpura nephritis. Journal of Clinical Dermatology, 1997, 26(3): 177-179.
42. Lu HP. Combined methotrexate with leflunomide for severe rheumatoid arthritis. Modern Journal of Integrated Traditional Chinese and Western Medicine, 2013, 22(18): 1979-1980.
43. Lu W, Xu DS, Wang WJ, et al. Leifengguan mixtures for 22 cases with rheumatoid arthritis. Shanghai Journal of Traditional Chinese Medicine, 1993, 9: 012. , 1993, 9: 012.
44. Lu YH. Tripterygium glycosides for the treatment of generalized eczema. Journal of Baotou Medical College, 2007, 23(4): 388-389.
45. Mao RY, Zhang YY, Sun WX, et al. Combined leflunomide with Tripterygium glycosides verse prednisone alone for IgA nephropathy. Chinese Journal of Integrated Traditional and Western Nephrology, 2009, 10(7): 604-606.
46. Meng LF. Tripterygium glycosides combined with Huoba Huagen tablets in the treatment of 38 patients with nephrotic syndrome. Journal of Guangxi Medical University, 2001, 6: 078.
47. Min J, Xiao MG, Gu ZH, et al. Combined Tripterygium glycosides with leflunomide for severe rheumatoid arthritis. Pharmacology and Clinics of Chinese Materia Medica, 2012, 28(4): 106-108.
48. Pang XW, Li SY, Liu WS, et al. Minocycline plus Tripterygium glycosides for acne conglobate. Chinese Journal of Dermatology, 2003, 36(1): 54.
49. Qian YY, Li YT, Ao JH, et al. Adverse effects of Tripterygium glycosides after homotransplantation of the kidney. Chinese Journal of Urology, 1995, 16(11):690.
50. Qu Z. China Prednisone plus Tripterygium glycosides tablets in the treatment of blister tetter. Foreign Medical Treatment, 2009 (17): 93-93.
51. Ren XQ, Ding Y. Clinical study of Tripterygium glycosides induced lung injures. Lishizhen Medicine and Materia Medica Research, 2007, 18(5): 1207-1208.
52. Rong YH. Efficacy of Bushen Qushi Juanbi method rheumatoid arthritis. Journal of Medical Forum, 2007, 19(4): 297-298.
53. Shen JP, Zhou M, Yan LB, et al. Clinical efficacy of Tripterygium glycosides for erythema nodosum leprosum. Journal of Diagnosis and Therapy on Dermato-venereology, 2013, 20(3): 164-168.
54. Shen XF. Compound licorice plus Tripterygium glycosides in the treatment of patients with anaphylactoid purpura nephritis. Zhejiang Medical Journal, 2010 (11): 1709-1710.
55. Shen YZ, Wang JQ, Xu XM. Clinical efficacy of Tripterygium mixtures for psoriasis. Modern Journal of Integrated Traditional Chinese and Western Medicine, 2008, 17(23): 3597-3598.
56. Sun RH, Lin ZH. Zhengqing Fengtongning for 38 patients with rheumatoid arthritis. Shanghai Journal of Traditional Chinese Medicine, 1999 (2): 21-23.
57. Sun W, Zeng AP. Clinical trial of Shenyanling granule verses other Tripterygium preparations. Chinese Traditional Patent Medicine, 2001, 23(11): 801-804.
58. Sun XJ. Observation of effects of Tripterygium Wilfordii in Treating the Diabetic Nephropathy Proteinuria. Nanjing University of Chinese Medicine, 2012.
59. Tao XL, Sun Y, Shi YP, et al. Low dose of Tripterygium preparation for rheumatoid arthritis. Chinese Journal of Integrated Traditional and Western Medicine, 1990, 10(5): 289-291.
60. Zhong JT. Tripterygium glycosides tablets plus Compound Danshen injection for 41 cases with psoriasis vulgaris. Tianjin Pharmacy, 2011, 23(3): 42-43.
61. Wang BX, Yuan ZZ. Extract Tablets Chinese Journal of Integrated Traditional and Western Medicine, 1989, 9(7): 407-408.
62. Wang CH. Santeng Juanbi decoction for 72 cases of rheumatoid arthritis. Chinese Journal of Information on Traditional Chinese Medicine, 2005, 12(8): 68-69.
63. Wang HM. Clinical observation of Acitretin and Tripterygium preparations for Palmoplantar Pustulosis. Chinese Journal of Misdiagnostics, 2007, 7(13): 2966-2966.
64. Wang HW, Chen JH. Tripterygium glycosides combined with the small dose of prednisone in the treatment of nephrotic syndrome. Chinese Journal of Integrated Traditional and Western Nephrology, 2008, 9(2): 165-166.
65. Wang JD, Xu YY, Zhang LQ, et al. Compare Tripterygium glycosides combined with prednisone verse prednisone alone in the treatment of 41 elderly patients with primary nephrotic syndrome. Chinese Journal of Integrated Traditional and Western Nephrology, 2003, 4(3): 171-171.
66. Wang J, Chang BC, Yang P, et al. Clinical trial of combined leflunomide with prednisone for refractory nephrotic syndrome. Chinese Journal of General Practice, 2011, 9(8): 1192-1193.
67. Wang QW, Li LS, Zhang JH, et al. Clinical study of Tripterygium glycosides for IgA nephropathy. Jiangsu Medicine, 1991, 1(1): 7-7.
68. Wang SY. Efficacy and safety of compound Tripterygium wilfordii for active rheumatoid arthritis. Shandong University of traditional Chinese medicine, 2004.
69. Wang W, Zhang HL. Efficacy of combination of Cetirizine and Tripterygium wilfordii for chronic urticarial. Chinese Journal of Rural Medicine and Pharmacy, 2006, 13(8): 57-58.
70. Wang YF. Cyclophosphamide therapy for children with severe Henoch-Schonlein purpura nephritis. Chinese Journal of Misdiagnostics, 2007, 7(25): 6019-6020.
71. Wu B. Tripterygium glycosides tablets plus Valsartan for for patients with chronic glomerulonephritis. Health must read, 2013, 12(5):270-272.
72. Wu M, Xie W, Ma YC. Combination of Salazosulfamide with Tripterygium glycosides for older adults with elderly rheumatoid arthritis. Journal of Clinical Medicine in Practice, 2005, 9(5): 87-87.
73. Xu JG. Efficacy of Tripterygium glycosides in the treatment of patients with chronic renal insufficiency. Modern Medicine Journal of China, 2007, 9(3): 12-12.
74. Xu MZ. Prospective Controlled Study of Tripterygium Wilfordii plus Emodin and Benazepril In the treatment of patients with IgA Nephropathy. Nanjing University, 2003.
75. Xu QL. Cyclophosphamide therapy for 36 patients with Henoch-Schonlein purpura nephritis. Practical Journal of Medicine & Pharmacy, 2006, 23(9): 1070-1071.
76. Xu TM. Clinical trial of Tripterygium glycosides tablets for chronic nephritis. Zhejiang Practical Medicine, 2003, 8(6): 353-353.
77. Xu XZ, Wu X. Low dose of Tripterygium glycosides plus prednisone for 37 patients with purpura nephritis. Acta Academiae Medicinae Suzhou, 2003, 8(6): 353-353.
78. Xu ZX. Combined low dose of Tripterygium glycosides and Molecular Heparin for chronic glomerulonephritis. Journal of Internal Medicine, 2008, 3(1): 17-18.
79. Yan GP, Zhang XL, Hao Z, et al. Chinese herbal medicines in the treatment of 40 patients with rheumatoid arthritis. Journal of Hebei Medical University, 2000, 21(5): 299-300.
80. Yang HZ. Quantitative analysis of T lymphocyte subsets in atopic eczema patient with Tripterygium glycosides treatment. Chinese Journal of Integrated Traditional and Western Medicine, 2007, 27(7): 592-592.
81. Yang JH, Zhang JY. Efficacy of four herbal remedies for patients with primary glomerulopathy. Journal of Shandong University of Traditional Chinese Medicine, 2001, 25(5): 344-346.
82. Yang XH, Yu RF. in Clinical observation of 40 patients with primary acquired nephrotic syndrome. The Journal of Practical Medicine, 1995, 11(11): 725-726.
83. Yao WC, Nian HF. Compound Tripterygium wilfordii wine for 145 patients with ankylosing spondylitis. Chinese Remedies & Clinics, 2004, 4(1): 72-73.
84. Yao WC, Nian HF. Compound Tripterygium wilfordii wine for rheumatoid arthritis, a 392 cases trial. Chinese Journal of New Drugs and Clinical Remedies, 2004, 23(1): 35-37.
85. Ye TS, Li F, Xie WX, et al. Combined Abdominal acupuncture with Tripterygium wilfordii in the treatment of postmenopausal women with active rheumatoid arthritis. Chinese Archives of Traditional Chinese Medicine, 2009, 27(9): 1944-1946.
86. Ye ZZ, Zhuang JH. Long term effects of five DMARDs in the treatment of ankylosing spondylitis. Chinese Journal of Clinical Pharmacy, 2002, 11(2): 65-68.
87. Yin LP, Su J, Zhang P, et al. Clinical trial of Tripterygium glycosides for chronic allograft nephropathyhinese. Journal of Organ Transplantation, 2009 (9): 545-547.
88. Yuan LJ. Tripterygium wilfordii in the treatment of allergic purpura. Journal of Liaoning University of Traditional Chinese Medicine, 1999, 1(2): 110-111.
89. Zhang EL, Xing F, Wu WZ. Compound Tripterygium wilfordii in the treatment of 362 patients with rheumatoid arthritis. National Conference on Rheumatic Diseases of Integrated Traditional Chinese and Western Medicine, 2004.
90. Zhang JF, Bao WR. Clinical trial of 50 children with ankylosing spondylitis. Popular Healthy News, 2013 (11): 74-74.
91. Zhang L, Zhang AJ. Clinical observation Tripterygium glycosides plus mifepristone as adjuvant therapy for women after surgery for endometriosis. Hubei Journal of Traditional Chinese Medicine, 2011, 33(9): 50-51.
92. Zhang L, Zhang QY, Xiong B. Efficacy of Tripterygium Wilfordii for psoriasis vulgaris. Strait Journal of Preventive Medicine, 2007, 13(4): 106-106.
93. Zhang MJ, Jin S, Ma YZ, et al. Tripterygium total terpenoids tablets for rheumatoid arthritis: 106 cases clinical study. Nanjing University of Chinese Medicine, 1993 (2): 10-12.
94. Zhang MO, Wang YJ, Chen HY, et al. Total glucosides of paeony in the treatment of mesangial proliferative glomerulonephritis: a 109 cases controlled trial. National Conference on Nephrology of Integrated Traditional Chinese and Western Medicine, 1993 (2): 10-12.
95. Zhang R, Wu CL, Li SF, et al. Combined leflunomide with Tripterygium glycosides for the elderly with severe rheumatoid arthritis. Chinese Journal of Gerontology, 2011, 31(12): 2194-2196.
96. Zhang WZ, Wang SH, Wang M, et al. Long-term effects of Tripterygium Wilfordii Hook. F. on gonads of children. Journal of Clinical Pediatrics, 1994, 5: 016.
97. Zhang WZ, Wang SH. Long-term effects of Chlorambucil in children with Kidney diseases. Chinese Journal of Integrated Traditional and Western Medicine, 1996, 16(2): 113-114.
98. Zhang YM. Efficacy of Tripterygium Wilfordii Hook. F., Erythromycin and compound licorice in the treatment of psoriasis. Chinese Journal of Postgraduates of Medicine, 2012, 35(003): 57-59.
99. Zhao DC. Different Dosage of Tripterygium glycosides for refractory nephrotic syndrome. Anhui Medical Journal, 2012, 33(10): 1377-1379.
100. Zhao TX. Tongbi decoction for 50 cases with rheumatoid arthritis. Journal of Practical Traditional Chinese Medicine, 2006, 21(10): 588-588.

**Prospective single arm studies**

1. Bu JA. Methotrexate plus Chinese herbal medicine for the treatment of rheumatoid arthritis: a study of 22 cases. Chinese Medicine of Factory and Mine, 2008, 21(4): 432-433.
2. Chen F, Luo HM. Clinical study of Tripterygium glycosides in the treatment of mesangial proliferative glomerulonephritis. Guide of China Medicine, 2005 (5): 118-118.
3. Chen JG, Wu AP, Jiang JX, et al. Combined leflunomide with Tripterygium glycosides for refractory nephrotic syndrome. Clinical Focus, 2010 (9): 814-816.
4. Chen K, Yang XY. Four drugs combination therapy in the treatment of 10 patients with gangrene. Southern China Journal of Dermato-Venereology, 1996, 3(3): 10-11.
5. Chen XZ, Wu MQ. Combination treatment for the treatment of rheumatoid arthritis. China Tropical Medicine, 2006, 6(3): 472-473.
6. Chen ZM. Effectiveness and safety of Tripterygium glycosides for glomerulonephritis: a study of 95 cases. Journal of Henan University, 2002, 3: 001.
7. Chi JM, Han BH, Yuan TT, et al. Intermittent administration of Tripterygium glycosides tablets for recurrent nephrotic syndrome. Chinese Journal of Traditional Medical Science and Technology, 2007, 14(2): 105-105.
8. Ding D, Hao XP. Huangteng tablets for endometriosis: 30 cases report. Hubei Journal of Traditional Chinese Medicine, 1994, 16(4): 21-22.
9. Ding N, Fan L, Yang XH. Tripterygium preparations in the treatment of 10 patients with uveitis. Jiangsu Journal of Traditional Chinese Medicine, 1989, 10(4): 15-17.
10. Dong XG. The effect of Tripterygium glycosides for cholesterin levels of diabetic nephropathy patients. Journal of Qiqihar Medical College, 2006, 27(4): 416-417.
11. Du SH, Wang HD, Li X, et al. Combined leflunomide with small dose of steroid therapy for idiopathic membranous nephropathy. Modern Chinese Doctor, 2011, 49(24): 99-101.
12. Du XL. Adverse effects of Leiluozhi tablets in the treatment of rheumatoid arthritis. The 4^th^ National Conference on Rheumatic Diseases of Integrated Traditional Chinese and Western Medicine, 2000.
13. Du XL, Liu Y. Adverse effects of Leiluozhi tablets for patients with rheumatoid arthritis. Shandong Journal of Traditional Chinese Medicine, 2001, 20(7): 403-404.
14. Fan XZ, Chen Y. Side effects of Tripterygium wilfordii Hook f in the treatment of auto-immune disease. Chinese Journal of Integrated Traditional and Western Nephrology, 2001, 2(2): 83-85.
15. Feng YZ, Lin XX, Niu LN, et al. Tripterygium wilfordii Hook f for the treatment of rheumatoid arthritis: a study of 100 cases. 1989, 4: 010.
16. Fu P, Lei M, Zhou L, et al. Double dosage of Tripterygium glycosides for the treatment of renal proteinuria. Journal of West China University of Medical University, 2002, 33(2): 318-319.
17. Fu WL. Integrated Traditional Chinese and Western medicine in children with persistent hematuria. Shanghai Journal of Traditional Chinese Medicine, 2002, 36(5): 20-21.
18. Fu YF, Liang JN, Shi YP. Tripterygium wilfordii Hook f for endometriosis: a study of 26 cases. Jiangsu Journal of Traditional Chinese Medicine, 1993, 4: 023.
19. Gao DM, Li HL, Liu LM. Cyclosporin and Tripterygium glycosides for thrombocytopenia. A Guide of China Medicine, 2013, 11(33): 373-374.
20. Gao JT. Tripterygium glycosides for steroid dependent asthma. Journal of Fujian Medical University, 1994, 28(2): 162-163.
21. Gao P, Bai HQ. Clinical observation of 12 Children with pustular psoriasis. China Practical Medical, 2011, 6(1): 160-160.
22. Gao WP, Wang AX. Intermittent administration of high dose of Tripterygium glycosides for glomerulonephritis patients with proteinuria. Chinese Medicine of Factory and Mine, 2001, 14(2): 106-107.
23. Gao WB, Wang YW, Zhu YH. Tripterygium glycosides for patients after homotransplantation of the kidney. The Journal of Practical Medicine, 2007, 23(5): 733-734.
24. Gao X, Wang XM. Effectiveness of Tripterygium Wilfordii for psoriasis vulgaris. Chinese Journal of Modern Drug Application, 2009, 3(18): 145-146.
25. Gao YP, Jin ZM, Chen DP, et al. Tripterygium glycosides for hysteromyoma. Acta Universitatis Medicinalis Secondae Shanghai, 2001, 21(2): 137-140.
26. Gu JH, Zhu CF, Wang W, et al. The effects of Lei Gong Teng on reproductive hormones[J]. Journal of traditional Chinese medicine, 2001, 21(1): 50-51.
27. Guo JL, Yuan SX, Wang XC, et al. Tripterygium wilfordii tincture in the treatment of rheumatoid arthritis. Journal of Medical Research, 1980, 8: 003.
28. Guo JL, Yuan SX, Wang XC, et al. Tripterygium wilfordii Hook f in rheumatoid arthritis and ankylosing spondylitis. Preliminary report[J]. Chinese medical journal, 1981, 94(7): 405.
29. Guo QY, Ding Y. Yigan Shenbao plus Tripterygium glycosides for Hepatitis B virus infection in children. Journal of Sichuan of Traditional Chinese Medicine, 2003, 21(6): 63-65.
30. Guo QY, Ding Y. Combined Tripterygium glycosides, Lotensin and heparin in children with Henoch-Schönlein Purpura Nephritis. Collection of the 3^rd^ Paediatrics National Conference, 2007.
31. Guo SL, Wu FX, Li X. Tripterygium wilfordii Hook f for chronic glomerulonephritis. Hebei Journal of Traditional Chinese Medicine, 1995, 17(6): 29-30.
32. Guo TH. Combined methotrexate, leflunomide with Tripterygium glycosides for rheumatoid arthritis. Journal of Chinese Modern Medicine, 2009, 6(2): 96-98.
33. Guo ZW, Liu N. Integrated Traditional Chinese and Western Medicine in the treatment of 12 patients with refractory nephrotic syndrome. Journal of New Chinese Medicine, 1995, 27(4): 26-27.
34. Hu WX, Tang Z. Double dosage of Tripterygium glycosides in the treatment of primary nephrotic syndrome. Chinese Journal of Nephrology Dialysis & Transplantation, 1997, 6(3): 210-214.
35. Jiang X. Clinical observations on the use of the Chinese herbTripterygium wilfordii Hook for the treatment of nephrotic syndrome [J]. Pediatric Nephrology, 1994, 8(3): 343-344.
36. Jin HB, Yu CH, Xiong NN. Safety of Tripterygium glycosides for chronic glomerulonephritis: a study of 74 cases. Journal of Nanjing University of Traditional Chinese Medicine, 1982, 1: 009.
37. Lai CY, Chao CX, Huang T, et al. Tripterygium glycosides for children with mild and severe bronchial asthma. Journal of Practical Pediatrics, 1987, 2(1): 28-29.
38. Lan YR, Liu SF, Xiang Q. Mayi preparation combination in the treatment of 184 patients with rheumatoid arthritis. Journal of Modern Clinical Medicine, 1996, (1):3-4.
39. Lao YN, Zhang ZL. Tripterygium glycosides for patients with rheumatoid arthritis. New Chinese medicine, 1985, (2):72.
40. Lao ZY. Adverse effects of long term use of Tripterygium glycosides for rheumatoid arthritis. Chinese Journal of New Drugs and Clinical Remedies, 1997, (1):36-36.
41. Li F. Liuwei Dihuang decoction plus Tripterygium glycosides for 35 cases with IgA nephropathy. Shandong Journal of Traditional Chinese Medicine , 2001, 20(11):676-677.
42. Li H, Zhong JM, Zhou HY, et al. Adverse effects of Tripterygium glycosides for r glomerulonephritis. Adverse Drug Reactions Journal, 2000, 2(1):25-27.
43. Li JP, Feng YH. Double dosage of Tripterygium glycosides for the treatment of refractory nephrotic syndrome. Journal of Nantong University, 1999, (2):210-210.
44. Li RL. Juvenile Tripterygium Wilfordii Hook. F. in the treatment of 104 patients with Juvenile rheumatoid arthritis. Pharmacology and Clinics of Chinese Materia Medica, 1992, (1):38-40.
45. Li XF. Risk factors for adverse outcomes of Tripterygium glycosides for children with kidney disease. Henan University of Traditional Chinese Medicine, 2008.
46. Li XQ, Yang LX, Wang XL, et al. U.S. Integrated Chinese and Western medicine for the treatment of 47 cases with ankylosing spondylitis. Chinese International Journal of Traumatology, 2010, (1):39-40.
47. Li XW. Tripterygium glycosides tablets for Henoch-Schönlein Purpura Nephritis in 50 children. Jiangsu Medicine, 1987, (12): 664-665.
48. Li XY, Xu N, Li H, et al. Triple cocktail therapy in the treatment of 22 patients with idiopathic membranous nephropathy. Annual congress of Zhejiang society of nephrology, 2012.
49. Li XQ, Gao M, Huang FF. Tripterygium glycosides for the treatment of diabetic nephropathy. China Practical Medical, 2009, 4(18):178-179.
50. Li Y. Tripterygium glycosides plus methotrexate for the treatment of rheumatoid arthritis. Modern Medicine & Health, 2007, 23(5):639-640.
51. Liang XW. Tripterygium glycosides in the treatment of 28 patients with asthma. Practical Clinical Journal of Integrated Traditional Chinese and Western Medicine, 1995, (4):247-247.
52. Lin DH, Zhuang YZ. Four drugs combination therapy in the treatment of 136 patients with gangrene. Clinical Medicine of China, 2000, 16(6):471-472.
53. Liu GL, Gao YF, Xia ZK, et al. Jiangsu Medicine, 1998, 19(12):13-14.
54. Liu GL, Xia ZK, Gao YF, et al. 1999, (3):264-265. Double dosage of Tripterygium glycosides plus Telmisartan for the treatment of children with primary nephrotic syndrome. Journal of Shanxi Medical University, 1999, (3):264-265.
55. Liu GL, Gao YF, Xia ZK, et al. Tripterygium glycosides in the treatment of children with nephrotic syndrome. Journal of Medical Postgraduate, 2003, 16(7):518-520.
56. Liu J. Zhou CY, Huang T, et al. Adverse outcomes of Tripterygium glycosides for chronic nephritis proteinuria. Herald of Medicine, 2010, 29(4):543-545.
57. Liu SX, Yang YY, Huang T, et al. Tripterygium glycosides with prednisone for 13 adults with purpura nephritis. Qinghai Medical Journal, 1994, (3):39-39.
58. Liu XN, Chen XQ, Chen SP, et al. Tripterygium glycosides plus methotrexate for severe psoriasis. The 14^th^ annual meeting of Chinese society of dermatology, 2008.
59. Liu XG, Zheng YH. Tripterygium glycosides plus acitretin for severe psoriasis. China Journal of Leprosy and Skin Diseases, 2005, 21(10):824-825.
60. Liu Y, Liu ES. Tripterygium tablets and herbal decoction for 20 patients with rheumatoid arthritis. The 4^th^ National meeting of Tripterygium Wilfordii Hook. F. 2004.
61. Liu ZZ, Hu WX, Zhang HT, et al. Tripterygium glycosides plus prednisone for lupus nephritis. Chinese Journal of Nephrology Dialysis & Transplantation, 2008, 17(6):512-516.
62. Long ZH, Yin ZY. Tripterygium preparation for Psoriatic arthritis. Beijing Journal of Traditional Chinese Medicine, 1983, (1):27-28.
63. Lu XX, Wang Y, Dong ZQ, et al. Prednisone plus Tripterygium glycosides tablets in the treatment of thrombocytopenia. Journal of Experimental Hematology, 2004, 12(1):98-100.
64. Luo HP. Thalidomide decoction in the treatment of 10 cases with Behcet's disease. Journal of Jiujiang University, 2002, 17(1):41-41.
65. Luo SJ. Tripterygium glycosides tablets for renal proteinuria: 100 cases. Chinese Pharmaceutical Affairs, 2005, 19(10):630-631.
66. Mei SS. Adverse effects of long term use of Tripterygium glycosides for rheumatoid arthritis. Heilongjiang Medicine and Pharmacy, 1997, (5):72-73.
67. Ni HL, Liu BL. Tripterygium and short wave combination therapy for rheumatoid arthritis. Journal of navy medicine, 2010, 31:37-38.
68. Niu FL, Yin SF, Niu FC, et al. Tripterygium Wushe San in the treatment of 606 patients with psoriasis vulgaris. Collection of Conference of China Association of Traditional Chinese Medicine, 1997.
69. Piao B. Tripterygium glycosides for the treatment of patients with chronic idiopathic urticarial. Journal of Misdiagnostics, 2010, 10(31):7720-7720.
70. Qian SZ, Hu YZ, Tong JS, et al. The effect of Tripterygium preparations for adult males. Chinese Journal of Andrology, 1989,3 (3):129-132.
71. Qian XH. , 2000, 16(6):380-381. Double dosage of Tripterygium glycosides plus Telmisartan for the treatment of primary nephrotic syndrome. Zhejiang Medical Journal, 2000, 16(6):380-381.
72. Qin WZ. Tripterygium preparations for 60 patients with systemic lupus erythematosus. Chinese Traditional Patent Medicine, 1982, (9):23-25.
73. Qu BZ, Ying LP, Li YH, et al. Tripterygium tablets in the treatment of 81 patients with Erythema multiform. Chinese Traditional Patent Medicine, 1983, 3: 017.
74. Rong Y, Hu WX. New Tripterygium glycosides treatment for idiopathic membranous nephropathy. Chinese Journal of Nephrology Dialysis & Transplantation, 1998, 7(5): 409-414.
75. Shi FX, Sun La, Zhu Y, et al. Tripterygium glycosides for Graves' eye disease. Journal of New Medicine, 1990, (9):471-472.
76. Shi ZQ, Ding M, Zhu M, et al. Clinical observation of Tripterygium glycosides combined with the small dose of prednisone in the treatment of senile primary nephrotic syndrome. Chinese Journal of Integrated Traditional and Western Nephrology, 2009, 10(3): 242-244.
77. Ethyl acetate extracts of Tripterygium Wilfordii Hook. F. in the treatment of 270 patients with rheumatoid arthritis. Pharmacology and Clinics of Chinese Materia Medica, 1989, 5(3): 40-42.
78. Song YJ, Ding WZ. The extracted of Tripterygium Wilfordii Hook. F. aerial part for severe rheumatoid arthritis. Pharmacology and Clinics of Chinese Materia Medica, 1993, (4):44-45.
79. Su QS, Xue YL. Long term use of Tripterygium glycosides for rheumatoid arthritis: 7 cases. Beijing Journal of Traditional Chinese Medicine, 1986, (6):32.
80. Sun YY, Cui YC, Jia Z, et al. Tripterygium Glycosides for 8 patients with renal proteinuria. Chinese Journal of Laboratory Diagnosis2012, 16(8):1464-1465.
81. Tang Y, Zhang X, Su PP. Adverse effects of Tripterygium preparations used in dermatology: an analysis of 163 cases. Journal of Clinical Dermatology, 2003, 27(6): 377-377.
82. Tao XD, Lu HR. Integrated Traditional Chinese and Western Medicine in the treatment of severe rheumatoid arthritis. The Journal of rheumatology, 2013, 2(9): 41-42.
83. Tao XL, Cush J J, Garret M, et al. A phase I study of ethyl acetate extract of the Chinese antirheumatic herb Tripterygium wilfordii hook F in rheumatoid arthritis[J]. The Journal of rheumatology, 2001, 28(10): 2160-2167.
84. Wang DC. Oral administration of Tripterygium Wilfordii for psoriasis vulgaris. Journal of Nursing Science, 1994, 9(1):20-21.
85. Wang JH. Tripterygium plus methotrexate for psoriasis: 35 cases. Southern China Journal of Dermato-Venereology, 1997, 4(4): 20-20.
86. Wang JH, Liang JP. Tripterygium glycosides for treating four patients with chronic glomerulonephritis. Jiangxi Journal of Traditional Chinese Medicine, 1987, 6: 014.
87. Wang JZ, Wang ZF, Chi JM, et al. Tripterygium glycosides tablets for patients with renal proteinuria. Heilongjiang Medical Journal, 2005 (12): 19.
88. Wang LF, Wang RJ. Tripterygium glycosides tablets Tripterygium glycosides tablets for lupus nephritis for lupus nephritis. Journal of Clinical Medicine, 2008, 27(11): 26-27.
89. Wang P. Tripterygium and sodium houttuyfonate tablets for the treatment of 30 children with eczema. Chinese Community Doctors, 2012, 14(13): 241-241.
90. Wang TM. Tripterygium glycosides for the treatment of hysteromyoma. Journal of Henan Medical College for Staff and Workers, 2007, 19(5): 458-460.
91. Wang YX, Ren K, Lu Y, et al. Tripterygium glycosides in the treatment of 20 cases with primary nephrotic syndrome. China Journal of Traditional Chinese Medicine , 2003, 6: 011.
92. Wei RH. Adverse outcomes of Tripterygium glycosides for kidney disease. Journal of Jinggangshan Medical College, 1998, 1.
93. Wei XJ, Zhu PJ. Chinese Journal of New Drugs and Clinical Remedies, 1988, 7(6): 371-372.
94. Weng YH. Tripterygium wilfordii syrup in the treatment of 39 patients with systemic lupus erythematosus. Zhejiang Journal of Traditional Chinese Medicine 2004, 39(4): 158-158.
95. Wu AH. The Tripterygium glycosides and Benazepril Hydrochloride Tablets in the treatment of Henoch-Schönlein Purpura Nephritis in 50 children. The Chinese and foreign health abstract, 2012, 09(15).
96. Wu GQ, Wu WY, Jin L, et al. Clinical and experimental study of Tripterygium preparations for psoriasis. The 4^th^ National meeting of Tripterygium Wilfordii Hook. F. 2004.
97. Wu GW, Xu WJ, Wang ZL, et al. Double dosage of Tripterygium glycosides plus Telmisartan for the treatment of renal proteinuria. The 3^rd^ Zhejiang middle west science and technology forum, 2006.
98. Wu QZ. Tripterygium glycosides tablets for recurrent nephrotic syndrome. Hainan Medical Journal, 2006, 17(3): 58-58.
99. Wu ZL, Yin ZW. Diyin tablets and Binghuang Fule in the treatment of patients with psoriasis. Strait Pharmaceutical Journal, 2007, 19(10): 98-99.
100. Xi ZP, Feng YL. Tripterygium glycosides tablets for perimenopausal patients with abnormal uterine bleeding: 50 cases study. Nei Mongol Journal of Traditional Chinese Medicine, 2010, 29(19): 8-9.
101. Xia N, Wang HJ, Ren B. Double dosage of Tripterygium glycosides plus Telmisartan for the treatment of Nursing Journal of Chinese People's Liberation Army, 2003, 20(5): 22-24.
102. Xie BG, Zhong W, Meng SL. Safety of Tripterygium glycosides tablets. Guangxi Medical Journal, 2002, 24(4):576-579.
103. Xie DF, Shen JH, Pei JF, et al. Thalidomide combined with Tripterygium Glycosides in the treatment of Behcet's disease. Chinese Journal of Integrated Traditional and Western Medicine, 1983, 6: 016.
104. Xu JY, Zhao CG, Wang K. Double dosage of Tripterygium glycosides plus Telmisartan for the treatment of primary nephrotic syndrome. Chinese Journal of Integrated Traditional and Western Nephrology, 2004, 5(3): 159-160.
105. Xu JZ, Huang SB, Xiao ZY, et al. Chinese Journal of Primary Medicine and Pharmacy, 1996, (3):11-13.
106. Xu JZ, Zeng QY, Huang SB, et al. Adverse effects of Tripterygium glycosides. Chinese Journal for Clinicians, 2001, 6: 042.
107. Xu LJ, Zhao J. Integrated Chinese and Western medicine for the treatment of IgA nephropathy. The Journal of Practical Medicine, 1992, 4: 023.
108. Xu SJ. Tripterygium wilfordii for autoimmune hemolytic anemia: a pilot study. Zhejiang Journal of Integrated Traditional Chinese and Western Medicine, 1994, 2 (2):29-29.
109. Yan BY, Zhai GJ, Jiang MZ. Tripterygium wilfordii for 165 cases with rheumatoid arthritis. Journal of First Military Medical University, 1985.
110. Yang RM, Qiu H. Tripterygium tablets plus Cetirizine for chronic urticarial. Journal of Gannan Medical University, 2005, 24(6): 753-753.
111. Yang Y, Jin LY, Cao L. Effectiveness of Tripterygium tablets for renal diseases. Ningxia Medical Journal, 2002, 24(11): 684-684.
112. Yao HF, Yu QS. Tripterygium tablets for subacute thyroiditis. Chinese Traditional Patent Medicine, 1994, 16(10): 26-26.
113. Yao WC, Nian HF. Tripterygium wine for rheumatoid arthritis: 3 year follow up. Ningxia Medical Journal, 2007, 29(5): 453-454.
114. Yao WC, Nian Y. Tripterygium wilfordii wine for ankylosing spondylitis: two year follow up. The 7^th^ National Conference on Rheumatic Diseases of Integrated Traditional Chinese and Western Medicine, 2008.
115. Yao YX, Geng KM, Fang J. Double dosage of Tripterygium glycosides plus Telmisartan for the treatment of renal diseases. Journal of Chinese Physician, 2005, 7(2): 278-279.
116. Yao YX. Adverse effects of double dosage of Tripterygium glycosides for the treatment of renal diseases. integrated Chinese and Western medicine for the treatment of
117. Ye B, Zhang SZ. Tripterygium glycosides for the treatment of thrombocytopenia: 20 cases. Zhejiang Clinical Medical Journal, 2000, 2(1): 54-54.
118. Ye HM, Chen WL, Chen WZ. Double dosage of Tripterygium glycosides for the treatment of renal failure. The Journal of Practical Medicine, 2011, 27(2): 288-290.
119. Ye LY, Lin YT, Chen XM, et al. Tripterygium Wilfordii Hook. F. in the treatment of 24 patients with refractory nephrotic syndrome. Fujian Journal of Traditional Chinese Medicine, 1988, 5: 028.
120. Yi ZJ, Zhang YQ. Tripterygium Wilfordii Hook. F. against pain: 40 cases report. Shanghai Journal of Traditional Chinese Medicine, 1987, 2: 032.
121. Yu D Y. Clinical observation of 144 cases of rheumatoid arthritis treated with glycoside of Radix Tripterygium Wilfordii[J]. Journal of traditional Chinese medicine, 1983, 3(2): 125-129.
122. Yu DY. Long term use of Tripterygium glycosides for rheumatoid arthritis. Jiangsu Medicine, 1987, 13: 661-661.
123. Yu JB, Wang HJ, Li F. Tripterygium glycosides plus Porcine Anterior Pituitary and Adrenal Cortex Extracts Injection for psoriasis. The Chinese Journal of Dermatovenereology, 1992, 3: 061.
124. Yu LL, Hu YQ, Wu LD. Double dosage of Tripterygium glycosides plus Telmisartan for the treatment of Heilongjiang Nursing Journal, 1999, 11: 034.
125. Yuan HM. Modern Journal of Integrated Traditional Chinese and Western Medicine, 2002, 11(14): 1390-1391.
126. Yuan HY. Tripterygium glycosides in the treatment of 87 cases of psoriasis vulgaris. China Xiaoyi, 2001, 15(1): 46-47.
127. Yuan JX. Tripterygium tablets in the treatment of 36 patients with rheumatoid arthritis. Clinical Journal of Traditional Chinese Medicine, 2006, 18(3): 295-296.
128. Yuan XL. Tripterygium glycosides plus Acitretin for 32 patients with palmoplantar Pustulosis. Journal of Dermatology and Venereology, 2008, 30(4): 38-39.
129. Yuan ZW, Zhou ZS, Zhong M. Fengshi wine and Shenjin decoction advanced rheumatoid arthritis. Lishizhen Medicine and Materia Medica Research, 2008, 19(6): 1479-1480.
130. Yuan ZW, Zhong M, Zhou ZS. Tripterygium tablets and herbal medicine for patients with rheumatoid arthritis. The Journal of rheumatology, 2012, 1(4): 8-10.
131. Yuan ZW, Zhong M. Tripterygium tablets and herbal medicine for children with ankylosing spondylitis. The Journal of Cervicodynia and Lumbodynia, 2008, 29(1): 39-41.
132. Yuan ZW. Tripterygium wine plus Huoxue Zhuangjin tablets for 120 patients with ankylosing spondylitis. Journal of Practical Traditional Chinese Internal Medicine, 2010 (3): 68-69.
133. Zhang EL. Tripterygium glycosides tablets for 68 patients with ankylosing spondylitis. Fujian Journal of Traditional Chinese Medicine, 1996, 27(2): 12-13.
134. Zhang HZ, Guo XX, Liu SP. Vitamin AD combined with Tripterygium Glycosides in the treatment of Behcet's disease. Journal of Changzhi Medical College, 1998, (4):292-293.
135. Zhang JZ. Erythromycin plus Tripterygium glycosides in the treatment of psoriasis vulgaris. Zhejiang Journal of Integrated Traditional Chinese and Western Medicine, 2013, 23(5): 373-374.
136. Zhang JZ. Roxithromycin and Tripterygium glycosides in the treatment of psoriasis vulgaris. The 4th National meeting of Tripterygium Wilfordii Hook. F.2004.
137. Zhang J. Tripterygium Wilfordii Hook. F. wine as adjunct therapy for 80 patients with rheumatoid arthritis. Clinical Journal of Chinese Medicine, 2012, 4(14): 75-76.
138. Zhang JT. Tripterygium Wilfordii Hook. F. in the treatment of 12 patients with Erythema multiform. Fujian Medical Journal, 1985, 6: 003.
139. Zhang KZ. Tripterygium wilfordii wine for rheumatoid arthritis. Ningxia Medical Journal, 1992, 5: 021.
140. Zhang PL, Yang HT. Adverse hematologic events of Tripterygium glycosides for rheumatic disease. The Journal of Practical Medicine, 2001, 17(9):817.
141. Zhang XA, Liu XL, Shao DB. Double dosage of Tripterygium glycosides plus Telmisartan for the treatment of senile primary nephrotic syndrome. Chinese Journal of Ethnomedicine and Ethnopharmacy, 2012, 21(16): 109-110.
142. Zhang YY, Jiang LJ. Tripterygium powder preparation for 33 patients with rheumatoid arthritis. Acta Academiae Medicinae Zunyi, 1983, 4: 015.
143. Zhang ZL. Tripterygium capsules for rheumatoid arthritis and ankylosing spondylitis. Shanghai Journal of Traditional Chinese Medicine, 1988, 11: 020.
144. Zhao C, Chen ZR, Zhou MT. Adverse outcomes of Tripterygium wilfordii for primary nephrotic syndrome. Clinical Focus, 1998, 13(24): 1131-1132.
145. Zhao JM, Li YJ, Li GM. Double dosage of Tripterygium glycosides for the treatment of primary nephrotic syndrome. Chinese Journal of Coal Industry Medicine, 2011, 14(4): 518-519.
146. Zheng CS. Clinical study of Tripterygium Wilfordii bilayer tablets. The 4th National meeting of Tripterygium Wilfordii Hook. F.
147. Zheng DH, Yu PZ, Yu SS, et al. Tripterygium wilfordii for chronic glomerulonephritis: 53 cases report. Chinese Journal of Practical Internal Medicine, 1982.
148. Zhong M, Yuan ZW. Compound Tripterygium wilfordii wine for 100 patients with rheumatoid arthritis. China's Naturopathy, 2006, 14(9): 62-64.
149. Zhong M. Compound Tripterygium wilfordii wine for 60 patients of ankylosing spondylitis. 6^th^ National Conference on Rheumatic Diseases of Integrated Traditional Chinese and Western Medicine
150. Zhou ZS, Liang RR. Combined prednisone with Tripterygium glycosides for adult purpura nephritis. Guangdong Medical Journal, 1999, 20(6): 472-473.
151. Zhu CL, Guan G. Low dose of Tripterygium glycosides plus methotrexate and chondroitin sulfate for the treatment of rheumatoid arthritis. Chinese Community Doctors, 2012, 14(12): 135.
152. Zhu HW, Zhu C, Lu W, et al. Long term use of Tripterygium glycosides for chronic glomerulonephritis. Acta Universitatis Medicinalis Secondae Shanghai, 2002, 22(2): 543-544.
153. Zhu JX. Thymic peptide and Tripterygium glycosides for the treatment of rheumatoid arthritis. Hebei Medicine, 1999, 5(4): 7-8.
154. Zhu QW, Guo FZ, Li YZ, et al. 8893-TW Liniment and Tripterygium for the treatment of 23 patients of rheumatoid arthritis. Fujian Medical Journal, 1990, 3: 044.
155. Zhuang WC, Li XM, Han XH, et al. Tripterygium glycosides plus vindesine for the treatment of ITP. Jiangsu Medicine, 2006, 32(2): 184-185.
156. Dermatology of Chinese Academy of Medical Sciences. Tripterygium glycosides in dermatology. Acta Academiae Medicinae Sinicae, 1979, 1(2):136-138.

**Case series**

1. Bi KO. Tripterygium wilfordii Hook F acute induced renal failure: an analysis of 20 cases. Chinese Journal of Modern Applied Pharmacy, 2000, 17(6): 502-502.
2. Bo FJ, Yu XG. Tripterygium glycosides induced amenorrhea: a clinical analysis of 11 women. Journal of Practical Medical Techniques, 2004, 11(2): 188-188.
3. Chen LY, Cai H, Yu DY. Tripterygium wilfordii Hook F toxicity: an analysis of 10 cases. Jiangsu Medicine, 1987, 13(12): 667-667.
4. Fan CC, Song L. Adverse outcomes of Tripterygium wilfordii Hook F for renal diseases: an analysis of 34 cases. Chinese Journal of Practical Internal Medicine, 1995, 15(6): 348-349.
5. Gu CX,Tao XL, Ge GS, et al. Tripterygium glycosides tablets induced amenorrhea. Acta Academiae Medicinae Sinicae, 1989, 11(2): 151-153.
6. Hu ZX. Tripterygium Wilfordii Hook. F. poisoning: an analysis of 18 cases. Clinical Focus, 1997, 12(21): 1002-1002.
7. Jiang YS, Zhao SP. Tripterygium wilfordii Hook F induced renal failure: an analysis of 15 cases. Journal of Central South University of Technology, 1987, 3: 028.
8. Liu YF, Deng HY. Clinical analysis of Tripterygium glycosides induced lung injures. Research of Traditional Chinese Medicine, 1997, 13(5): 20-20.

**Case reports**

1. Bi KB, Jia YL. Renal failure induced by Tripterygium wilfordii Hook F root overdose: a case report. China Journal of Chinese Materia Medica, 2000, 25(3): 191-191.
2. Cao GJ, Ruan XD. Erythema nodosum induced by Tripterygium glycosides tablets: a case report. China Journal of Chinese Materia Medica, 1994, 19(12): 759-759.
3. Cao Y. Hematopoietic disorders induced by Tripterygium preparations: two case reports. Chongqing Medicine, 1995, 24(2): 127-127.
4. Chen GJ, Chen YK. Orchitis induced by Tripterygium glycosides tablets: a case report. Adverse Drug Reactions Journal, 2000, 2(1): 56-56.
5. Chen N. Atrophy of ovary following oral Tripterygium wilfordii Hook F: a case report. Acta Chinese Medicine and Pharmacology, 1993, 2: 023.
6. Chen YH. Cardiogenic shock after Tripterygium wilfordii Hook F decoction: three case reports. Fujian Medical Journal, 1988, 1: 019.
7. Cui XM, Liu X. Tripterygium glycosides tablets related bone marrow suppression: a case report. Pharmacology and Clinics of Chinese Materia Medica, 1988, 1: 019.
8. Dang RY. Fixed drug eruption induced by Tripterygium glycosides tablets: a case report. The Chinese Journal of Dermatovenereology, 1995, 3.
9. Feng D. Aplastic anemia after long term use of Tripterygium tablets: a case report. Jiangsu Journal of Traditional Chinese Medicine, 1991, 8: 028.
10. Feng GA, Guo NJ, Dong XB, et al. Aplastic anemia after the use of Tripterygium glycosides tablets: case reports and literature review. Journal of Clinical Hematology, 2005, 18(1): 42-44.
11. Fu BH. Serious adverse effects of whole and root of Tripterygium wilfordii Hook F. The 4^th^ National meeting of Tripterygium Wilfordii Hook. F. 2004. Gao QY, Wang BY. Shock following oral Tripterygium wilfordii Hook F: a case report. Journal of Henan college of Traditional Chinese Medicine, 2002, 17(3): 49-50.
12. Gao QY, Wang BY. Case report: treatment of overdose of Tripterygium decoction induced shock. Journal of Henan college of Traditional Chinese Medicine, 2002, (3):49-50.
13. Hao XC. Drug eruption induced by Tripterygium glycosides tablets: a case report China Journal of Chinese Materia Medica, 1993, 18(3): 182-182.
14. He MC, Li XB, Zhang Y, et al. Hepatorenal damage induced by Tripterygium preparations: three case reports. Adverse Drug Reactions Journal, 2006, 8(2): 136-137.
15. Huang GZ, Li L, Liu L, et al. Pathology of Tripterygium wilfordii Hook F toxicity: a study of four cases with autopsy report. Chinese Journal of Integrated Traditional and Western Medicine, 2009 (2).
16. Jin DM. Pseudomembranous colonitis after use of Tripterygium tablets: a case report. Chinese Journal of New Drugs and Clinical Remedies 1993, 12(2): 94-94.
17. Jin LP, Han BL. Bradycardia induced by Tripterygium preparations: 7 cases. Chinese Journal of Cardiovascular Rehabilitation Medicine, 2001, 10(3): 267-268.
18. Ke KZ. Lung injury: a case report. Practical Clinical Medicine, 2009, 1(7).
19. Li BC, Ke RL, Zhong Y, et al. Agranulocytosis: case report. Academic Journal of Second Military Medical University, 1991, 12(1): 95-96.
20. Li HY, Mi J. Tripterygium glycosides tablets: a complication of its misuse in overdose. Journal of Changchun University of Traditional Chinese Medicine, 2010, 26(6): 892-892.
21. Li HG, Xue L, Zhou JQ. Atrioventricular block induced by Tripterygium preparations: a cases report. Clinical Misdiagnosis & Mistherapy, 1998, 4.
22. Li XY, Zhang JY. Aplastic anemia after the use of Tripterygium glycosides tablets: a case report. Chinese Journal of Misdiagnostics, 2010, 10(22): 5537-5537.
23. Lin P. Fatal aplastic anemia after Tripterygium tablets: a case report. China Journal of Chinese Materia Medica, 1992, 17(10): 630-630.
24. Lin WM, Zhang Y. Pancytopenia induced by Tripterygium glycosides tablets: a case report. Journal of the Fourth Military Medical University, 2006, 27(18): 1707-1707.
25. Liu GC. Tripterygium tablets-induced facial pigmentation: case report. Journal of Heze Medical College, 2004, 16(1): 19-19.
26. Liu GX. Bone marrow suppression induced by Tripterygium Wilfordii Hook. F.: 2 case reports. Journal of Chengdu University of Traditional Chinese Medicine, 1997, 20(3):44-45.
27. Liu L. Tripterygium glycosides tablets induced pain of small bones of the hands and feet: 6 cases. Heilongjiang Journal of Traditional Chinese Medicine, 2000, (3):15.
28. Liu XS, Zou AY, Shen L, et al. Tripterygium glycosides tablets induced lung injury: a case report. Tianjin Pharmacy, 2008, 20(1): 29-30.
29. Lu F, Zhang DX, Tian SM. Aplastic anemia associated with ulcerative colitis: 2 case reports. Shanghai Medical Pharmaceutical Journal, 1995, 7: 018.
30. Lu XH, Chen QY, Zhong HD, et al. Tripterygium glycosides tablets induced granulocytopenia: 9 cases. Chinese Journal of Hospital Pharmacy, 1993, 13(12): 559-559.
31. Ma LP. Tripterygium induced organ failure: a case. Chinese Journal of Integrated Traditional and Western Medicine in Intensive and Critical Care, 1997, 4(3): 116-116.
32. Pan GG, Gao WP. Drug allergy induced by Tripterygium glycosides tablets. Journal of Pharmaceutical Practice, 2003, 21(1): 52-52.
33. Pei F, Yang XL, Tan LY, et al. A case of allergic urticaria caused by oral administration of Tripterygium Wilfordii. Medical Journal of the Chinese People's Armed Police Forces, 2003, 1: 014.
34. Qian WJ. Tripterygium Wilfordii Hook. F. induced alopecia: 2 cases report. Journal of Dermatology and Venereology, 1982: 46-47.
35. Shen SY, Zha JQ. Tripterygium Wilfordii Hook. F. wine poisoning: 3 cases. Journal of Huazhong University of Science and Technology, 1979, 2: 025.
36. Shi JH, Wang MJ, Liu XD. Tripterygium Wilfordii Hook. F. induced amenorrhoea: 2 cases. Traditional Chinese Drug Research & Clinical Pharmacology, 2003, 22(10): 635-636.
37. Shou XZ. Tripterygium Wilfordii Hook. F. induced acute renal failure. Zhejiang Journal of Integrated Traditional Chinese and Western Medicine, 1999, 9(4):276-277.
38. Su JS. Tripterygium Wilfordii Hook. F. induced gastrointestinal symptoms: 2 cases reports. Journal of Jiangxi University of Traditional Chinese Medicine, 2000 (S1): 15-15.
39. Tian XS. Tripterygium glycosides tablets associated with ptosis. Chinese Journal of Modern Applied Pharmacy, 1993, 5: 038.
40. Tu X, Mao LM, Cheng XX. Acute renal failure induced by compound Tripterygium wilfordii: case report. Zhejiang Practical Medicine, 2001, 6(6): 52-52.
41. Wan DM, Peng ZG. Case report: Hypothyroidism induced by Tripterygium glycosides tablets. Journal of China clinical medicine, 2006, (66):5389.
42. Kang JR. Hemolytic Uremic Syndrome: a case report. Ningxia Medical Journal, 1982, 3: 012.
43. Wang JX, Lai YW. Tripterygium Wilfordii Hook. F. poisoning. Liaoning Journal of Traditional Chinese Medicine, 2007, 34(1): 99-100.
44. Wang JY, Wang Z, Zhao J. Tripterygium Wilfordii Hook. F. induced lung injury: a case report. Chinese Journal of Integrated Traditional and Western Nephrology, 2003, 4(2): 81-81.
45. Wang L, Zhou HL. Tripterygium glycosides tablets induced leukopenia: case report. Chinese and Foreign Medical Research, 2012, 10(30): 27-27.
46. Wang XJ, Tan X. Tripterygium Wilfordii Hook. F. induced thrombopenia: a case report. Jilin Medical Information, 1994 (12): 22-22.
47. Wang YN, Xu ZG. Tripterygium Wilfordii Hook. F. poisoning: 2 cases. Lishizhen Medicine and Materia Medica Research, 2000, 11(8): 757-757.
48. Fang JY, Cheng ZZ, Zhang LY, et al. One case of Tripterygium Wilfordii Hook. F. toxicity. Acta Academiae Medicinae Wannan, 2000, 11(8): 757-757.
49. Wu GH. Tripterygium glycosides induced acute agranulocytosis: case report. Zhejiang Journal of Integrated Traditional Chinese and Western Medicine, 1997, 2: 045.
50. Wu TJ. Oral administration of Tripterygium Wilfordii induced Restless leg syndrome: case report. Chinese Journal of Integrated Traditional and Western Medicine, 1993, 13(2): 93-93.
51. Xu JY. Tripterygium glycosides induced dysspermia: a case report. Chinese Journal of Hospital Pharmacy, 2012, 32(021): 1777-1778.
52. Xu Z, Yu FY, Fan FB, et al. Aplastic anemia after the use of Tripterygium glycosides tablets: 2 case reports. Zhejiang Journal of Integrated Traditional Chinese and Western Medicine, 2006, 15(11): 704-705.
53. Yang LX. Obstructive jaundice caused by Tripterygium Wilfordii Hook. F. report of one case. Acta Academiae Medicinae Zunyi. 1996, 19:258.
54. Yang SR, Yang XZ, Wang SL. Death associated with Long term use of Tripterygium Wilfordii Hook. F. poisoning: 2 cases. Shanghai Journal of Traditional Chinese Medicine, 1993, 2(3): 38.
55. Yang WW. Tripterygium Wilfordii Hook. F. induced thrombopenia: a case. Chinese Journal of Clinical Pharmacy, 2000, 9(3): 189-189.
56. Zhang HY, Zhang Y. Tripterygium Wilfordii Hook. F. induced lung injury: 2 case reports. Journal of Practical Hepatology, 1999 (3): 140-140.
57. Zhang Q. Tripterygium Wilfordii Hook. F. induced leukemoid reaction: report of one. Qinghai Medical Journal, 1997, 27(10): 60-60.
58. Zhang QX, Song M, Yang YX. Tripterygium glycosides induced dysfunctional uterine bleeding. Chinese Journal of Pharmacoepidemiology, 2004, 13(2): 83-83.
59. Zhang SY. Tripterygium glycosides tablets induced premature ovarian failure. Sichun medicine, 1995, 16(3): 191-191.
60. Zhang YH, Huang GZ. Tripterygium wilfordii Hook F toxicity: a autopsy report. Journal of Wuhan medical college, 1985, 5: 030.
61. Zhao HL, Jin QF. Tripterygium glycosides tablets induced ulcer. Chinese Journal of Clinical Pharmacy, 1995, 3: 012. , 1985, 5: 030.
62. Zhao SP. Tripterygium induced organ failure: a case. Modern Journal of Integrated Traditional Chinese and Western Medicine, 2000, 7: 076.
63. Zhong FG, Qu ZG, Jin JH, et al. Aplastic anemia induced Tripterygium glycosides: 3 case reports. Chinese Journal of Gerontology, 2006, 26(8): 1107-1109.
64. Zhou FW. Tripterygium glycosides tablets-induced facial pigmentation: case report. Medical Journal of West China, 2009, 21(3): 493-493.
65. Zhou YF, Gu ZP, Piu HY. Tripterygium glycosides tablets induced lung injury: a case report. Chinese Journal of Clinical Pharmacy, 2002, 11(5): 103-103.
66. Zhou ZS, Liu PC. Tripterygium Wilfordii Hook. F. induced multiple organ system failure.Chinese Journal of Rural Medicine, 1992 (5): 38-39.
