# Supplementary material for: Safety Profiles of Tripterygium wilfordii Hook F: A Systematic Review and Meta-Analysis
Source: Front Pharmacol. 2016 Nov 8;7:402. doi: 10.3389/fphar.2016.00402 (PMC5099241; doi:10.3389/fphar.2016.00402)

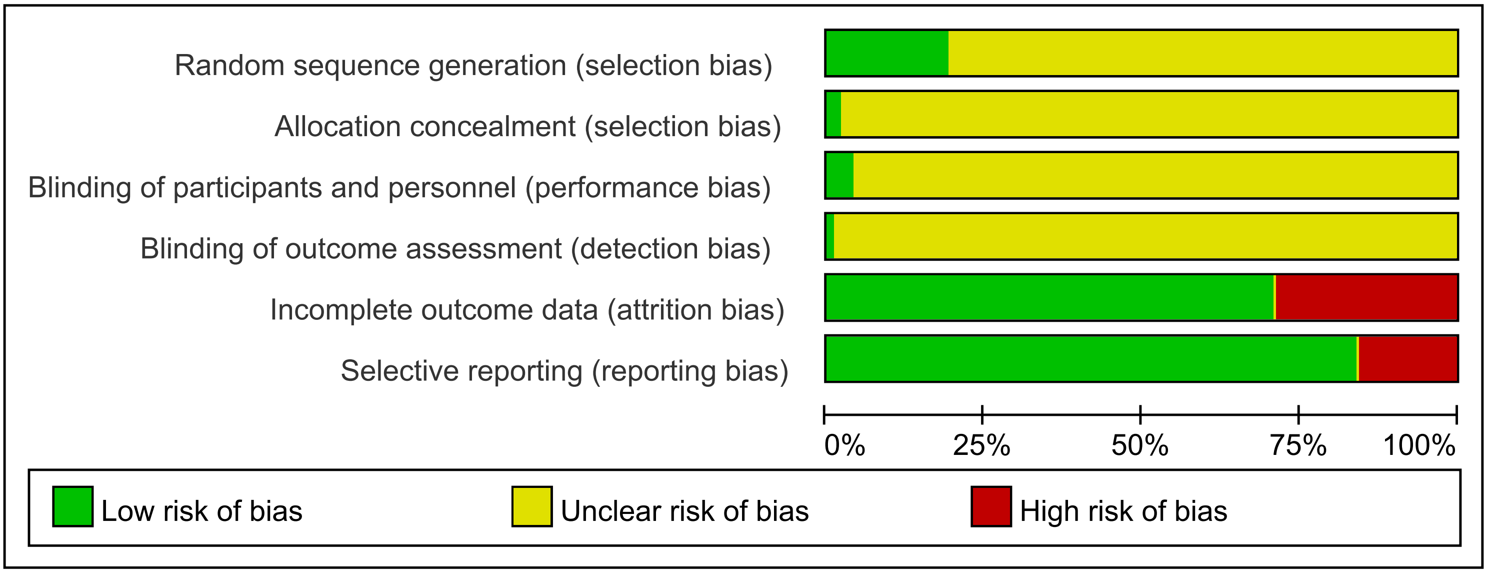
Random sequence generation (selection bias)
Allocation concealment (selection bias)
Blinding of participants and personnel (performance bias)
Blinding of outcome assessment (detection bias)
Incomplete outcome data (attrition bias)
Selective reporting (reporting bias)
0%
25%
50%
75%
100%
Low risk of bias
Unclear risk of bias
High risk of bias

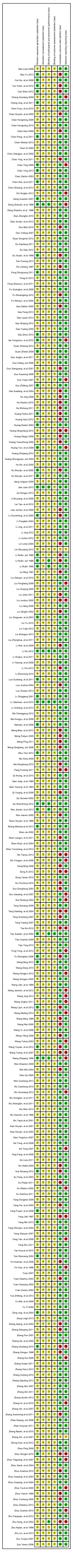

Supplement: Supplementary file 9 [file DataSheet9.pdf]
